# Phenotypic variation in neural sensory processing by deletion size, age, and sex in Phelan-McDermid syndrome

**DOI:** 10.1186/s11689-025-09642-4

**Published:** 2025-08-25

**Authors:** Melody Reese Smith, Elizabeth Berry-Kravis, Andrew Thaliath, Emily L. Isenstein, Allison R. Durkin, Jennifer Foss-Feig, Paige M. Siper, Charles A. Nelson, Lauren Baczewski, April R. Levin, Craig M. Powell, Stormi L. Pulver, Matthew W. Mosconi, Alexander Kolevzon, Lauren E. Ethridge

**Affiliations:** 1 Department of Psychology, University of Oklahoma, Norman, OK USA; 2 Stanford University, Palo Alto, CA USA; 3 Department of Pediatrics, Neurological Sciences & Biochemistry, Rush University Medical Center, Chicago, IL USA; 4 Rush University Medical Center, Chicago, IL USA; 5 Department of Brain and Cognitive Sciences, University of Rochester, Rochester, NY USA; 6 Psychiatry, Icahn School of Medicine at Mount Sinai, New York, NY USA; 7 Seaver Autism Center Department of Psychiatry, Icahn School of Medicine at Mount Sinai Hospital, New York, NY USA; 8 Boston Children’s Hospital, Boston, MA USA; 9 Harvard Graduate School of Education, Cambridge, MA USA; 10 Children’s National Hospital, Washington, DC USA; 11 Department of Pediatrics, University of Oklahoma Health Science Center, Norman, OK USA; 12 Clinical Child Psychology Program Schiefelbusch Institute for Life Span Studies, University of Kansas, Lawrence, KS USA; 13 Department of Pediatrics, Emory University, Atlanta, GA USA; 14 Department of Neurobiology, UAB School of Medicine, Birmingham, AL USA; 15 Department of Neurology, Boston Children’s Hospital, Boston, MA USA

## Abstract

**Background:**

Phelan-McDermid Syndrome (PMS) is a rare genetic condition characterized by deletion or mutation of region 22q13.3, which includes the *SHANK3* gene. Clinical descriptions of this population include severely impaired or absent expressive language, mildly dysmorphic features, neonatal hypotonia, developmental delays, intellectual impairments, and autistic-like traits including abnormal reactivity to sensory stimuli. Electroencephalography (EEG) has shown promise as a tool for identifying neurophysiological abnormalities in neurodevelopmental disorders. However, few EEG studies focused on sensory processing have been performed on this population. Thus, this study focuses on comparisons of event-related potential (ERP), event-related spectral perturbation (ERSP), and inter-trial coherence (ITC) between PMS and typically developing (TD) individuals in a standard auditory gating task measuring attenuation of neural activity to repetitive auditory stimuli.

**Methods:**

A total of 37 participants, 21 PMS (12 females, age range 8–18.6 years) and 16 TD individuals (8 females, age range 8.2–15.3 years) were included. Analysis consisted of a series of general linear models using a regional (frontal) and global (whole-head) approach to characterize neural activity between PMS and TD participants by age, sex, and group.

**Results:**

Most notably, individuals with PMS had delayed or low amplitude P50, N1, and P2 responses in frontal and whole-head analyses as well as poor frontal phase-locking to auditory stimuli for alpha, beta and gamma ITC, indicating impaired processing of stimulus properties. Additionally, individuals with PMS differed from TD by age in delta, theta, and alpha power, as well as frontal beta-gamma ITC, suggesting different developmental trajectories for individuals with PMS. Within PMS, larger deletion sizes were associated with increased auditory processing abnormalities for frontal P50 as well as whole-head P50 and N1.

**Limitations:**

This is one of the largest EEG studies of PMS. However, PMS is a rare genetic condition, and our small sample has limited statistical power for subgroup comparisons. Findings should be considered exploratory.

**Conclusions:**

Results suggest that participants with PMS exhibit auditory processing abnormalities with complex variation by deletion-size, age, and sex with congruency to impaired early recognition (P50), feature processing (N1), information integration (delta, theta), sensory processing and auditory inhibition (alpha), and inhibitory modulation of repeated auditory stimuli (beta, gamma). Findings may provide valuable insight into clinical characterization of sensory and speech behaviors in future studies.

**Supplementary Information:**

The online version contains supplementary material available at 10.1186/s11689-025-09642-4.

## Background

Phelan-McDermid Syndrome (PMS), or 22q13.3 Deletion Syndrome, is diagnosed in early childhood and characterized by a dysfunction in the distal portion of chromosome 22 with a breakpoint at 22q13.3. This dysfunction results from a point mutation or deletion of *SHANK3*, a gene which acts as a scaffolding protein in the postsynaptic density of excitatory glutamatergic synapses [1]. In PMS, only one copy of *SHANK3* is functional, reducing dendrites in the cortex and impairing synaptic transmission and plasticity [1, 2]. The clinical features of *SHANK3* deletion or mutation include severely impaired or absent expressive language, mildly dysmorphic features such as a bulbous nose, deep-set eyes, long eyelashes, an elongated skull, as well as neonatal hypotonia, developmental delays and intellectual deficits, along with autistic traits [1, 3]. Reports estimate that between 84% and 94% of individuals with PMS also meet diagnostic criteria for Autism Spectrum Disorder (ASD) [4, 5]. However, diagnosis of ASD in severely intellectually impaired and non-verbal patients is a subject of controversy.

Given its relatively recent discovery and approximately 3,000 known cases, little investigation has been done using electroencephalography (EEG) on individuals with PMS in the realm of autism-relevant traits like sensory reactivity and potential effects of age and sex on neural development. Prior work has demonstrated that EEG is a valuable tool for identifying neurophysiological abnormalities, such as auditory processing impairments, in neurodevelopmental disorders like ASD [6, 7], FXS [8, 9], and PMS [10, 11]. The EEG-based auditory tasks used in these studies have links to neurophysiological processes that provide a window into understanding potential auditory processing abnormalities in these clinical populations. Thus, this study uses a standard auditory EEG gating task to identify differences in auditory processing among individuals with PMS compared to typically developing individuals (TD). Differences were measured with event-related potentials (ERPs), event-related spectral perturbation (ERSP), and inter-trial coherence (ITC). Variables of interest included age (in months), sex (male or female), group (PMS or TD), and deletion size (in bases), with additional consideration of potential confounds related to EEG data collection.

## Foundational EEG research

### Event-related potentials

Auditory gating, and sensory gating in general, is a regulatory mechanism in which neural processing of redundant or unnecessary sensory information is attenuated. Gating is critical for directing the brain’s resources to relevant environmental stimuli and avoiding sensory overload [12] and can be used more generally as a method of measuring the strength of inhibitory pathways in the central nervous system [13] using event-related potentials (ERPs). Four ERP components are investigated as potential biomarkers of auditory processing abnormalities in PMS individuals: the P50, N1, P2, and N2. The P50 is a fronto-centrally organized positive ERP component that occurs around 50 milliseconds post-stimulus and represents the brain’s initial cortical registration of an auditory event. It is thought to occur as a mechanism by which we filter out redundant or trivial information to avoid information overload by attenuating the second of two identical auditory stimuli presented in close succession [14, 15]. Although primarily generated by the temporal lobe [16, 17], the P50 has an additional frontal lobe generator crucial to the gating response; impairments in gating may stem from abnormalities in this fronto-temporal interaction [17]. Following the P50 is the N1, or N100, which is a negative ERP component generally peaking around 90–200 milliseconds after stimulus onset. Its scalp topography is fronto-centrally organized in adults [18]. Overall, the N1 is associated with detailed processing of stimulus properties and in the case of auditory gating, may be further correlated with the detection of matches or mismatches between consecutive stimuli. Next, the P2 is a centrally organized positive deflection that reaches its peak amplitude around 100–250 milliseconds after stimulus onset [14]. The P2 reflects registration of stimulus quantities and object recognition, therefore recruiting more resources from the temporal, frontal, and parietal lobes in the case of an auditory stimulus. Two main generators of the P2 ERP are Heschl’s gyrus and the auditory association cortex among other areas [19]. Lastly, the N2 is a late component that is often strongest in school-aged children and may have the same or similar generators to later subcomponents of the N1 [20]. With a typical gating response, the amplitude and latency of the P50, N1, P2, and N2 are significantly reduced in response to a repeated identical auditory stimulus, due to recurrent inhibitory properties of the neural ensembles representing the stimulus properties and the reduced need to recruit additional resources for recently processed information.

PMS is primarily characterized by sensory hyporeactivity, contrasting some single-gene disorders associated with autism, such as Fragile X Syndrome [21]. Therefore, PMS individuals are expected to have lower amplitude ERPs than TD individuals. With dysfunction of *SHANK3*, protein scaffolds and signaling at the postsynaptic density of excitatory glutamatergic synapses are likely impaired, which disrupts synaptic transmission and hinders a neuron’s ability to effectively adapt to the environment [22]. Thus, PMS is also expected to show poor gating responses, indicative of reduced ability to filter out unnecessary sensory information, despite reduced overall response to auditory stimuli.

### EEG power and phase-locking

Additionally, mouse models with *SHANK3* deletion have found low delta, low alpha, and high gamma power associated with reduced open field activity, deficits in sensory responses, and deficits in reciprocal social interactions [23, 24]. In humans, gamma oscillations are likely generated by parvalbumin-positive (PV+) cells, which play a role in inhibition of glutamatergic cells. Having an overactive inhibitory framework via PV + cells may contribute to enhanced gamma in PMS [23]. Furthermore, metabotropic glutamate receptors (mGluR5) are present in PV + cells, and both are highly concentrated in the striatum. A mouse model with deletion of exons 4–22 of *SHANK3* showed that striatal mGluR5 is disrupted in PMS by reduced synaptic Homer1 protein [24]. Dhamne et al. (2017) theorizes that increased PV + cell activity acts as a protective mechanism against seizures by reducing excitatory output, as evidenced by their mouse model with reduced seizure susceptibility. Thus, this may explain both the abnormal gamma oscillations in *SHANK3* models, as well as the seizure susceptibility of many patients diagnosed with PMS.

The role of other EEG power bands in PMS symptomatology is less clear, though delta oscillations are often thought to reflect inhibitory control, which may suggest impaired inhibition in prior mouse models of PMS with low delta power [25]. The alpha (and theta) bands may reflect thalamocortical modulation, in which impairments in these bands lead to abnormal gating of thalamic neurons (or hippocampal function) [26] or abnormal modulation of other oscillations like beta and gamma [27], particularly in the context of an auditory habituation task.

Like power measures, phase-locking is a potential biomarker for PMS and ASD, given the involvement of phase synchrony in both synaptic plasticity and cortical development that are disrupted in both conditions [28]. Although phase-locking has not been examined directly in PMS, previous human research has shown positive phase-bias in resting state PMS alpha-gamma coupling in posterior electrodes with maximal gamma occurring at the falling phase of alpha for PMS and the rising phase of alpha for TD [10]. This finding reflects potential abnormalities in thalamocortical circuit function in PMS, which we hypothesize to be captured by phase-locking measures as well. Literature on phase-locking abnormalities during auditory gating tasks among individuals with neurodevelopmental disorders such as Fragile X Syndrome (associated with sensory hypersensitivities) and ASD suggest that impaired phase-locking may represent difficulty organizing inhibitory networks to synchronize oscillatory activity, which may cause abnormal neuronal refraction and auditory habituation [9]. Thus, impaired phase-locking in PMS may similarly reflect less synchronized, more hyposensitive networks that prevent proper neuronal refraction. Given these parallel lines of research, as well as the involvement of phase synchrony in PMS-relevant brain functions, we performed an exploratory analysis of phase-locking in delta through gamma bands. Additionally, consistent with murine models, we predict that PMS will have less delta, less alpha, and more gamma power than TD.

### Deletion size

Several group studies have found a positive correlation between deletion size and the number and severity of behavioral and clinical features of PMS, including developmental delays and absence of speech [1, 29–32]. Deletions and point mutations of *SHANK3* are known, single-gene causes of PMS, whereas larger deletions can be multigenic. The additive impact of deletions suggests that larger deletions affect a greater range of genes and may ultimately lead to variations in type and severity of PMS phenotypes [1, 31, 32]. PMS mouse models also demonstrate deletion-size dependent effects, in which phenotypes vary with the precise exonal region of deletion within SHANK protein domains [33–40].

Deletion size may be a mediating factor in the relationship between gating responses of individuals with larger deletions versus point mutations. Thus, it is expected that individuals with PMS will show abnormally low amplitude ERPs, longer latencies, high gamma and low power otherwise, reduced phase-locking, and less effective gating with larger deletion sizes. The genetic variation of mutations and deletions as a source of physiological variation may be an important factor to consider in future studies of PMS, particularly given the small sample sizes likely to be available for studies of this rare disorder.

### Age and sex effects

By age 5 in PMS, individuals generally show severely attenuated motor and sensory development, and discrepancies between performances of children with PMS versus their TD peers become increasingly apparent as both groups age. Therefore, we predict that PMS will show more developmentally delayed responses, characterized by lower amplitudes and longer latencies than TD, and that these differences will become more severe with increased age. Variations in severity of developmental delay may also present as variations in topographic organization, as scalp topographies for auditory ERP components have largely matured in distribution by age 8 but may present in more immature distributions for individuals with PMS.

Although the prevalence and symptomology of PMS appears to not differ between males and females, there are pre-existing sex differences in the ERP response to an auditory gating task for TD individuals. Females generally have higher amplitude ERPs but similar latencies as males [41]. We expect to see individuals with PMS follow the same pattern as the TD population. The most consistent time-frequency findings suggest TD females having higher resting beta than males [42, 43], however there are discrepancies in the field. Sex differences also vary by region, in which the developmental trajectory of males and females show EEG coherence peaks in different cortical regions from each other across time [44]. We therefore consider power differences by sex to be exploratory in this study, with differences most likely to occur in beta power.

### Clinical relevance

This study uses EEG to identify differences in sensory processing of an auditory gating task using event-related potentials (ERPs), event-related spectral perturbation (ERSP), and inter-trial coherence (ITC) among individuals with PMS and typically developing individuals (TD). EEG has previously been successful for identifying and understanding sensory impairment in individuals with neurodevelopmental disorders such as FXS and PMS [9–11, 45]. These EEG brain biomarkers have potential to advance both basic scientific and clinical research—e.g., by furthering our understanding of the neuropathological processes involved in PMS, as well as by enabling early identification of patients with PMS, or tracking the efficacy of treatments or preventatives in clinical trials.

## Method

### Participants

Fifty-six participants were recruited for this study from 4 sites (Rush Medical Center, Boston Children’s Hospital, UT Southwestern, and Mount Sinai), and data were analyzed at the University of Oklahoma. Inclusion criteria required participants to be between ages 3 and 21 with English as their primary language. Healthy TD participants were excluded if they had a reported history of a learning, developmental, psychiatric, or neurological disorder, seizures, or current psychotropic medication use. PMS diagnoses were verified via clinical reports of a point mutation at the *SHANK3* locus or chromosomal deletion encompassing the *SHANK3* gene. Because of the variable topographies and development of gating responses in children under age 8 [12] and lack of matched controls for PMS children < 8 years old, 18 participants < 8 years old were excluded from the current analyses (see the age distribution of the analysis dataset between PMS and TD in Supplemental Figs. 1–2 and within PMS in Supplemental Figs. 3–4). A separate age-specific study of PMS auditory processing biomarkers with a larger sample and equal distribution of ages under and over age 8 would better characterize children with PMS than the current study and could address concerns of confounding age and developmental stage with EEG regions of interest (e.g., mismatches between lateralized vs. central gating topographies in children vs. adults). 1 dataset was excluded for having excessive artifacts. 37 datasets entered analysis with 21 PMS (12 females, age range 8-18.6 years) and 16 TD (8 females, age range 8.2–15.3 years) (Table 1). 16 out of the 21 individuals with PMS were taking medications: 3 were receiving antipsychotics, 6 ADHD medications (e.g., stimulants, alpha-2 agonists), 6 anticonvulsants, 3 anxiolytics, and 4 antidepressants, including polydrug combinations (see Supplementary Table 1 for more details).

**Table 1:** Sample characteristics

**Sex**			**Group**		**System**	
	Male	Female	TD	PMS	EGI	BioSemi
Male	17	−	8	9	11	6
Female	−	20	8	12	13	7
TD	8	8	16	−	11	5
PMS	9	12	-	21	13	8
EGI	11	13	11	13	24	−
BioSemi	6	7	5	8	−	13
Age (mos.)	145 (42)	138 (37)	129 (26)	151 (45)	147 (43)	131 (30)
Trials	124 (27)	125 (27)	120 (28)	128 (25)	119 (27)	136 (23)

Note: Diagonal values are totals. For example, the intersection of Male row and Male column is 17, indicating that 17 out of 37 subjects are male. Other values are sample sizes, with the mean (standard deviation) of age and trials at the bottom

### Procedures

Prior to the EEG tasks, TD participants, or a parent or guardian for those under 18, completed informed consent, an eligibility sheet, and a demographic questionnaire including a medication log. Due to intellectual disability in the PMS group, the consent form was completed by the individual’s legal guardian. Depending on data collection site, participants underwent EEG using a 33-channel BioSemi, 128-channel BioSemi or 128-channel EGI brand EEG system. Phantom testing of the gating task prior to study initiation showed comparable ERP amplitude and adjusted latency data from each system type given the pre-processing steps described below. Regardless, system type was included in all analyses as a covariate. Participants performed an auditory gating task while EEG was recorded. Individuals listened through headphones to paired identical 5ms broadband noise bursts (75db) separated by a 500 ms inter-stimulus interval. Participants were instructed to listen but not respond to the sounds. Each participant listened to 150 stimulus pairs (S1, S2) with an inter-trial interval of 4,000 ms.

### Clinical and resting data

Clinical measures were collected in a parallel resting study [10] and correlated with the current EEG measures to address the relationship between behavioral phenotype and neurophysiological outcomes. Measures included the Vineland Adaptive Behavior Scales, 2nd edition: Survey Interview Form (Vineland-II) Communication subscale [46], the Autism Diagnostic Observation Schedule, 2nd Edition (ADOS-2) with Social Affect & Restricted Repetitive Behaviors (SARRB) scores [47], the Short Sensory Profile (SSP) total score and auditory filtering subscale [48], Social Responsiveness (SRS) total t-score [49], and IQ. Full-scale IQ was recorded from either the Stanford-Binet, 5th Edition (SB-5; [50]), Differential Ability Scales, 2nd Edition (DAS-II; [51]), the Mullen Scales of Early Learning (MSEL; [52]) Early Learning Composite or a calculated Developmental Quotient. Nonverbal IQ was obtained from the SB-5 and DAS-II (nonverbal reasoning score). For participants who received the Mullen, a nonverbal developmental quotient was calculated by dividing mean age equivalents on the visual reception and fine motor scales by chronological age.

### EEG analysis

**Fig. 1:**
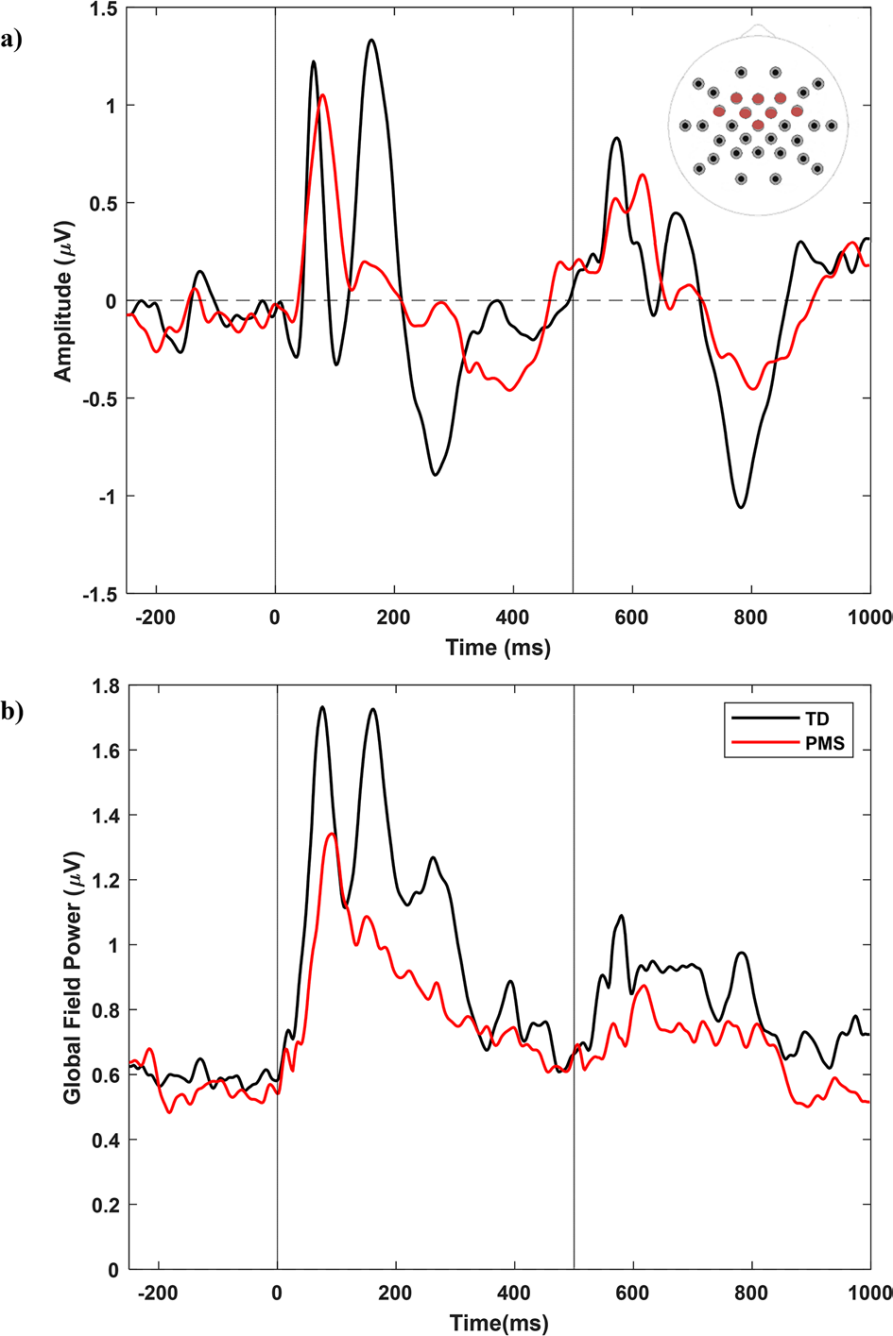
(**a**) Frontal and (**b**) Whole-head ERP Analysis, PMS (*n* = 21) vs. TD (*n* = 16) PMS, Phelan-McDermid Syndrome; TD, typically developing. Electrodes in a) include FC5, FC6, F3, FC1, F4, FC2, Fz, and Cz

Data were digitally filtered from 0.5 (12dB/octave slope; zero phase) to 50 Hz (24 dB/octave slope; zero-phase) with a notch filter at 60 Hz (width 2 Hz) and visually inspected. Bad sensors (no more than 5% of the total number of sensors) were interpolated using spherical splines in BESA 6.1 (MEGIS Software, Grafelfing, Germany). Since participant data came from a mixture of 33-channel BioSemi and 128-channel EGI nets, all data were re-montaged to a standard 33-channel average referenced montage (Fig. 1). Physiological and environmental artifacts were removed from the data using independent component analysis (ICA) in EEGlab 14.1.1. Epochs with remaining artifact over 120µV were excluded; all participants retained trial counts ≥ 59. Data were then downsampled to 500 Hz, epoched from 250ms before stimulus 1 to 500ms after stimulus 2 (−250 to 1000ms), baseline corrected, and averaged to create ERPs for each participant. We defined a post-stimulus time window for each ERP component of interest (P50: [40-120ms; 540-620ms]; N1: [90-200ms; 590-700ms]; P2: [120-250ms; 620-750ms]; N2: [200-350ms, 700-850ms]) based on peak amplitudes from grand averages. Peak amplitude and latencies were calculated as the most positive (P1, P2) or negative (N1, N2) deflections within relevant time windows using Matlab R2018a (The Mathworks, Natick, MA). Peaks were then verified manually to ensure accuracy in the order and timing of components, with 5 or fewer participants having a minimum or maximum latency 25ms beyond the expected ERP time windows. All but 1 extreme value came from participants with PMS. Verified ERP ranges including extreme values were as follows: P50: [42-156ms; 534-656ms]; N1: [84-246ms; 592-742ms]; P2: [124-300ms; 634-822ms]; N2: [178-402ms, 716-864ms]. Since these data involve participants with a neurodevelopmental disorder, we could not rule out the possibility that these extreme values were true processing delays. Thus, we opted to retain values exceeding our expected time windows.

**Fig. 2:**
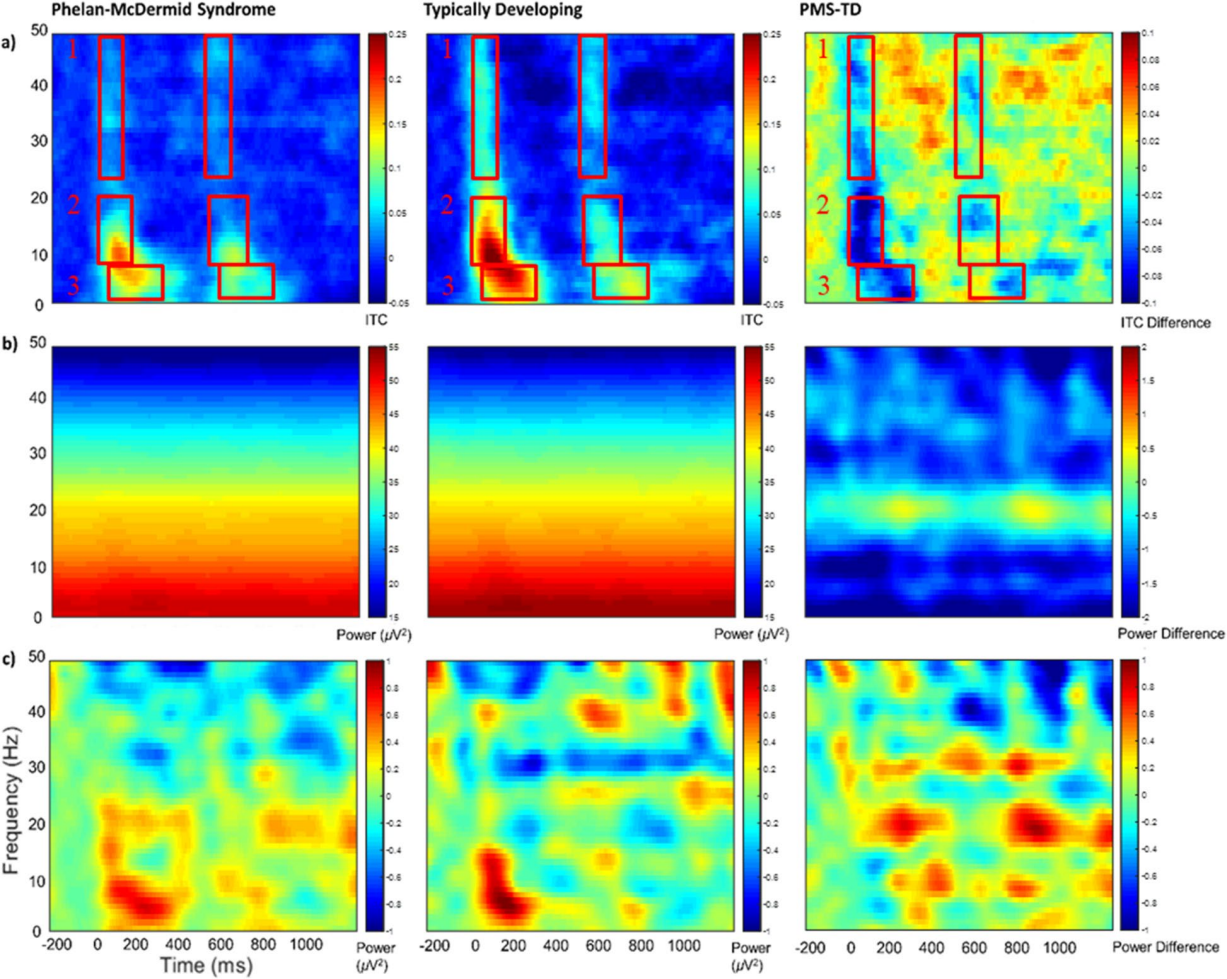
Frontal Time-Frequency Analysis. (**a**) Inter-trial phase coherence (unitless). ITC values were averaged across the indicated time and frequency ranges. (**b**) Single-trial power as event-related spectral perturbation (µV^2^). (**c**) Baseline-corrected single-trial power (µV^2^). Warmer colors indicate higher phase locking and power values, while cooler colors indicate lower values. PMS, Phelan-McDermid Syndrome; TD, Typically Developing

For time-frequency measures, epoch size was increased to −500 to 1500ms to allow padding for edge effects. Calculations were made with a modified Morlet wavelet with 1 Hz frequency resolution increasing from 1 to 20 cycles between 2 and 50 Hz. Power bands were specified according to standard literature: delta (2–3 Hz), theta (4–7 Hz), alpha (8–12 Hz), beta (13–30 Hz), gamma (30–50 Hz) and averaged within 500ms of stimulus 1 and 2. Inter-trial coherence (ITC) is a dimensionless measure calculated using time and frequency ranges, as denoted in Fig. 2, which represent stimulus processing in low (delta-theta), mid (alpha-beta), and high (beta-gamma) frequency bands. Since phase-locking is sensitive to trial-count, ITC values were corrected using the critical r value, sqrt[-(1/number of trials)^∗^log(0.5)]. ITC time and frequency bounds were based on the shape of the TD response in frontal analyses and applied to all ITC calculations (TD, PMS, frontal, whole-head). While analyzing ITC by frequency band would map more straightforwardly onto the power analyses, we believe our approach more optimally describes this ITC data. Here, we used functional rather than canonical frequency bands and time windows, focusing on how the frequencies function (in clusters) to build the ERP vs. using standard bands established for continuous or ongoing EEG. In a prior publication by our group [45], we saw a similar early high frequency component and later low frequency component. The ITC windows were chosen to best capture observed hotspots in the typically developing group, which varied in time and could be more easily averaged out with pre-set frequency band ranges (e.g., especially for the beta band here). The bifurcation between the lower boxes (e.g., in Fig. 2a) is intended to capture the time-shifted ITC in the delta-theta range.

### Frontal analyses

Frontal analyses were conducted on a subset of sensors (FC5, FC6, F3, FC1, F4, FC2, Fz, and Cz: Fig. 1) capturing the auditory EEG topography consistent with the literature [14, 17, 18]. Age, deletion size, and trials were mean-centered for interpretability and standardized scaling, since zero values for these variables do not exist in the dataset (Kraemer & Blasey, 2004). Then, a group LASSO protocol was performed in SAS 9.4 (SAS Institute, Cary, NC) using the proc glmselect command to determine the best configuration of equations for each dependent variable. Unlike other common variable selection methods for regression (e.g., forward selection, backward elimination, etc.), group LASSO optimizes models by selecting important *groups* of explanatory factors for predicting the outcome variable [53]. Specifically, group LASSO minimizes the residual sum of squares such that the sum of absolute values of regression coefficients is smaller than a specified constant and ensures that all levels of a categorical variable are maintained within a single variable, rather than separating the levels into unique predictors for model selection, as normal LASSO requires. This technique enables us to run complex, exploratory models despite having small sample sizes, which is powerful for studying rare neurodevelopmental diseases such as PMS. All data were standardized prior to computing group LASSO. Multicollinearity and normality of residuals were investigated using the proc reg procedure with variance inflation, tolerance, and collinearity eigenvalues as output.

The selection criteria included a default rho of 0.9 and a maximum of 20 steps, where each added variable maximized adjusted R^2^. For each dependent variable, the algorithm ran 500 times, and the most frequently occurring combination of variables was chosen for the final model to control for overfitting. Independent variables and covariates included in the group LASSO variable selection procedure included group, stimulus, age, sex, trials, EEG system, and all two-way interactions. Using output from group LASSO, regression models were run using proc glm on P50, N1, and P2 mean amplitudes and latencies for both stimuli in the PMS and TD groups with variables chosen by group LASSO. Group LASSO did not always include main effects alongside selected interactions; thus, main effects were added to GLM models when necessary to ensure proper variable hierarchy. Separate models were computed for the PMS group only including deletion size (bases) as an independent variable, with point mutations classified as a deletion size of 0.001 bases (1 kb). The same model selection methodology was used to calculate separate multiple regression models for time-frequency data with dependent variables including power in frequency bands from delta to gamma and ITC ranges for stimulus 1 and 2.

### Whole-head analyses

The global or whole-head averaging method utilizes global field power (GFP), which quantifies total amount of scalp activity using the mean of all absolute potential differences corresponding to the spatial standard deviation. Unlike the frontal analysis approach, GFP bypasses the issue of finding a set of electrodes that, when averaged, would encompass all participants’ brain activity during the auditory gating task. This is a concern as the impact of PMS on variation in the spatial distribution of neural signals is unknown. The dependent variables for this analysis included GFP (µV) and latencies (ms) at stimulus 1 and 2 for the P50, N1, and P2 as well as power and ITC as defined in the frontal analysis. With both techniques, we could determine whether the more commonly employed region-specific approach sufficiently captured differences in neural activation during the auditory gating task in a sample with potential for large topographical variation due to delayed or abnormal maturation of cortical sources. For GFP, 7 N1 peaks, 2 P2s, and 2 N2s could not be identified. Given the exploratory nature of this study, no multiple comparisons corrections were applied.

### Supplementary analyses

Exploratory univariable Wilcoxon-Mann-Whitney exact tests of frontal and whole-head group differences in PMS vs. TD stratified by auditory stimulus are included in the Supplementary Materials using the full sample (*N* = 37) and an age 8–12 restricted sample (*N* = 23) to determine whether the differences in age distributions between PMS and TD drive observed group effects.

## Results

Clinical correlations are presented in Table 2. Descriptive statistics for all EEG outcome variables are presented in Table 3. Age distributions of PMD and TD groups are presented in Supplemental Fig. 1. Statistical results of group LASSO and GLM procedures are summarized below. No issues with multicollinearity or non-normal residuals were found. For details on parameter estimates, see Tables 4–11. The following results are from frontal analyses; significant comparisons to whole-head findings are described in the section Frontal vs. Whole-Head.

**Table 2:** Frontal and Whole-head clinical correlations: PMS only

Dependent variable	SSP Total (*N* = 8)	SSP Auditory filtering (*N* = 9)	SRS Total tscore (*N* = 10)	ADOS SARRB (*N* = 10)	Vineland-II Comm. SS (*N* = 11)	Nonverbal IQ (*N* = 11)
**Frontal**						
P50A	**0.372**	0.284	− 0.027	− 0.059	**0.290**	0.220
N1A	− 0.223	− 0.059	0.229	0.164	0.146	0.080
P2A	**0.366**	0.030	− 0.251	− 0.184	− 0.134	− 0.037
N2A	− 0.262	− 0.061	0.057	0.017	0.150	0.112
P50L	**0.396**	**0.359**	**− 0.274**	− 0.230	0.201	0.218
N1L	0.167	0.234	− 0.063	0.054	0.065	− 0.002
P2L	0.198	0.213	− 0.020	0.242	**− 0.276**	**− 0.256**
N2L	0.238	0.015	− 0.134	− 0.032	− 0.145	− 0.077
Delta power	**− 0.327**	*−0.491	**− 0.302**	0.243	− 0.120	− 0.094
Theta power	− 0.286	**− 0.381**	− 0.238	**0.354**	**− 0.338**	**− 0.342**
Alpha power	− 0.280	− 0.250	− 0.036	**0.371**	− 0.167	− 0.162
Beta power	0.071	0.174	− 0.030	~ **0.422**	− 0.023	0.049
Gamma power	**0.310**	~ **0.415**	− 0.205	0.164	− 0.100	0.008
Beta-gamma ITC	− 0.057	0.138	*0.534	− 0.009	0.224	0.167
Alpha-beta ITC	~**−0.491**	~**−0.460**	*0.543	**− 0.328**	*0.510	*460
Delta-theta ITC	− 0.304	− 0.239	**0.311**	**− 0.352**	*0.525	~** 0.380**
**Whole-head**						
P50 GFP	~**0.470**	**0.309**	− 0.048	0.053	0.181	0.129
N1 GFP	*0.588	**0.344**	**− 0.348**	0.168	− 0.218	− 0.080
P2 GFP	*0.595	**0.301**	− 0.242	0.121	− 0.131	0.005
N2 GFP	**0.369**	0.213	− 0.072	0.176	**− 0.272**	− 0.243
P50L	**0.413**	~**0.413**	− 0.180	− 0.002	0.107	0.092
N1L	0.073	0.132	0.006	− 0.029	0.126	0.061
P2L	0.234	0.211	− 0.094	0.068	− 0.148	− 0.126
N2L	0.245	− 0.003	− 0.051	**− 0.281**	− 0.046	0.007
Delta power	− 0.137	− 0.203	**− 0.317**	*0.456	***−0.656	**−0.621
Theta power	− 0.208	− 0.252	**− 0.329**	*0.533	**−0.578	**−0.559
Alpha power	− 0.083	− 0.203	**− 0.338**	*0.501	*−0.505	*−0.485
Beta power	0.149	0.053	− 0.166	*0.463	**− 0.287**	− 0.199
Gamma power	0.167	**0.303**	− 0.151	**0.298**	− 0.200	− 0.107
Beta-gamma ITC	**0.414**	~** 0.434**	− 0.018	− 0.056	− 0.196	− 0.195
Alpha-beta ITC	**0.375**	0.199	− 0.030	0.052	~**−0.360**	− 0.239
Delta-theta ITC	− 0.018	0.044	**0.302**	~**−0.395**	0.022	0.049

All values are non-parametric Spearman’s rho correlations. The bold highlights potentially clinically relevant cells in which p <.25. ~p <.10, *p <.05, **p <.01, ***p <.001. A, amplitude; L, latency; GFP, global field power; ITC, intertrial coherence

**Table 3:** Descriptive statistics for ERP and Time-Frequency variables

Group	Type	Variable	Stimulus_F1_	Stimulus_F2_	Stimulus_W1_	Stimulus_W2_
TD	Amplitude	P50	1.80 (1.70)	1.36 (0.97)	1.85 (1.16)	1.39 (0.76)
		N1	−1.04 (1.52)	−0.55 (0.79)	1.71 (0.78)	0.97 (0.36)
		P2	1.85 (1.19)	0.94 (0.82)	1.86 (0.69)	1.26 (0.39)
		N2	−1.40 (0.64)	−1.32 (0.67)	1.47 (0.45)	1.14 (0.39)
	Latency	P50	68.9 (13.3)	89.4 (21.5)	68.0 (13.0)	87.4 (25.1)
		N1	112 (19.1)	139 (32.6)	114 (22.3)	138 (35.1)
		P2	176 (34.5)	185 (31.9)	169 (20.3)	183 (25.4)
		N2	269 (27.9)	288 (33.2)	267 (26.4)	284 (31.7)
PMS	Amplitude	P50	1.60 (1.02)	1.21 (0.76)	1.56 (0.71)	1.25 (0.48)
		N1	−1.01 (0.98)	−0.54 (0.56)	1.38 (0.82)	0.83 (0.39)
		P2	1.06 (0.95)	0.65 (0.60)	1.27 (0.68)	0.84 (0.41)
		N2	−0.97 (0.86)	−1.03 (0.67)	1.03 (0.66)	0.96 (0.50)
	Latency	P50	87.9 (29.2)	98.4 (31.5)	87.8 (21.9)	100 (30.3)
		N1	156 (47.8)	166 (39.9)	155 (46.1)	165 (36.6)
		P2	216 (54.1)	222 (45.4)	212 (50.3)	221 (45.0)
		N2	297 (66.4)	292 (41.2)	292 (63.4)	294 (42.2)
TD	Power	Delta	53.6 (2.81)	53.4 (2.89)	45.8 (3.02)	45.5 (3.01)
		Theta	52.3 (2.76)	51.9 (3.05)	45.4 (2.85)	45.1 (2.85)
		Alpha	48.5 (2.39)	48.3 (2.65)	43.5 (2.43)	43.1 (2.50)
		Beta	40.3 (2.07)	40.3 (2.07)	37.8 (1.68)	37.6 (1.70)
		Gamma	25.4 (3.23)	25.6 (2.94)	28.1 (1.88)	28.3 (1.67)
	ITC	Beta-Gamma	0.08 (0.07)	0.06 (0.06)	0.02 (0.03)	0.01 (0.02)
		Alpha-Beta	0.17 (0.11)	0.07 (0.07)	0.03 (0.03)	0.01 (0.02)
		Delta-Theta	0.17 (0.12)	0.11 (0.10)	0.03 (0.05)	0.03 (0.03)
PMS	Power	Delta	51.6 (4.37)	51.5 (4.24)	44.7 (2.91)	44.9 (3.08)
		Theta	50.7 (4.68)	50.4 (4.70)	44.1 (2.90)	44.1 (2.85)
		Alpha	46.9 (4.61)	46.7 (4.66)	42.4 (2.78)	42.3 (2.80)
		Beta	39.6 (4.78)	39.6 (5.05)	37.3 (2.20)	37.3 (2.49)
		Gamma	24.2 (4.92)	24.3 (5.20)	28.5 (2.86)	28.6 (3.28)
	ITC	Beta-Gamma	0.03 (0.05)	0.03 (0.03)	0.01 (0.03)	0.01 (0.02)
		Alpha-Beta	0.09 (0.08)	0.06 (0.04)	0.01 (0.02)	0.01 (0.02)
		Delta-Theta	0.13 (0.11)	0.10 (0.09)	0.03 (0.04)	0.01 (0.03)

Note: Table values are means (standard deviations). Amplitudes are measured in μV, latencies in ms, and power in dB. F, frontal; W, whole-head; TD, typically developing; PMS, Phelan-McDermid Syndrome

**Table 4:** Frontal ERP results: between PMS and TD

Variable	P50A (µV)	P50L (ms)	N1A (µV)	N1L (ms)	P2A (µV)	P2L (ms)	N2A (µV)	N2L (ms)
Group		**17.73		***44.08		***38.94		
Stim		***41.19		***50.50	**−0.630			
Age		−0.067		~−0.299			**0.006	
Sex		~ 12.06		~ 19.70	**−0.606			
Trials		−0.138		*−0.611				
System		5.978		8.805				
Group*Trials				~ 0.711				
Stim*Sex		~−19.74		*−30.30				
Stim*System		***−44.72		**−47.41				
Age*Sex		0.196		0.296				
Age*Trials		0.001						
System*Trials		**0.746		~ 0.855				
**Omnibus F**	F(1, 73) = 126	F(11, 62) = 5.30	F(1, 73) = 44.9	F(11, 62) = 6.27	F(2, 71) = 8.93	F(1, 72) = 14.80	F(1, 72) = 7.05	F(1, 73) = 2816
**Model** ***p*-value**	0.000	0.000	0.000	0.000	0.000	0.000	0.010	0.000
**Mean**	1.48	87.1	−0.78	146	1.09	202	−1.16	287
***R***	0.000	0.484	0.000	0.527	0.201	0.170	0.089	0.000
**Univariable: Stim 1**	−0.3 (−0.9, 0.4)	**8 (−4, 22)**	0.1 (−0.4, 0.8)	*30 (0, 70)	**−0.5 (−1.2, 0.1)**	5 (−20, 36)	**0.4 (−0.1, 0.9)**	23 (−24, 62)
**Univariable: Stim 2**	−0.1 (−0.6, 0.5)	2 (−16, 18)	0.2 (−0.2, 0.5)	−6 (−36, 18)	−0.1 (−0.6, 0.4)	**−24 (−46, 0)**	**0.3 (−0.1, 0.7)**	**−24 (−54, 10)**

Note: Table of parameter estimates. Each column represents a group LASSO model of an EEG outcome variable. Parameter estimates less than 0.001 were shortened to 0.000 to save space. ~*p* <.10, *p <.05, **p <.01, ***p <.001. Variables with no symbol were retained by group LASSO but not significant in the final model. A non-significant model means that independent variables did not improve model fit over an intercept-only model. Only variables chosen by group LASSO are presented. Reference groups are as follows—Stimulus: Stimulus 1, Sex: Male, Group: TD, System: EGI. A, amplitude; L, latency; Stim, stimulus; TD, typically developing; PMS, Phelan McDermid Syndrome. The univariable rows indicate the median difference and 95% confidence interval between PMS and TD for each EEG outcome variable to either the first or second auditory stimulus (for additional details on the univariable analyses, see Supplemental Table 2). For these univariable results, negative values are lower for PMS vs. TD, and positive values are higher for PMS vs. TD. The bold highlights potentially clinically relevant cells in which *p* <.25. ~*p* <.10, *p <.05, **p <.01, ***p <.001

### Main analysis: PMS vs. TD

In frontal analyses, individuals with PMS had significantly longer P50, N1, and P2 latencies than TD individuals across auditory stimuli, suggesting delayed auditory processing of stimulus characteristics in PMS (Tables 3 and 4; Fig. 1). Main effects also showed weaker frontal alpha-beta and beta-gamma ITC for individuals with PMS compared to TD (Table 5; Fig. 2a), which similarly suggest impaired auditory processing in PMS. Power variables highlighted a group difference by age, in which delta, theta, alpha power, and beta-gamma ITC had greater age-related increases for PMS, suggesting an abnormal developmental trajectory for PMS compared to TD within these EEG features (Tables 5 and 6; see Supplemental Fig. 2 for an example visualization). More specifically, power and high-frequency phase-locking decreased with age for TD individuals, while individuals with PMS showed an opposite and potentially over-compensatory pattern with excessively low power at younger ages and excessively high power at older ages. Additionally, a sex*group interaction showed that delta through beta power was weaker for females with PMS than for TD males (Table 5). This interaction effect showed similar levels of power for controls and male PMS; PMS females had a stark reduction in delta through beta power by comparison. Notably, there were sex-based differences in deletion size among PMS males and females, in which females tended to have larger deletions (Wilcoxon-Mann-Whitney exact *p* =.009).

**Table 5:** Frontal Time-Frequency results: between PMS and TD

Variable	Delta (µV^2^)	Theta (µV^2^)	Alpha (µV^2^)	Beta (µV^2^)	Gamma (µV^2^)	Beta-gamma (ITC)	Alpha-Beta (ITC)	Delta-Theta (ITC)
Group	−0.048	0.066	−0.259	0.472		*−0.028	*−0.038	
Stim							***−0.064	
Age	*−0.046	~−0.039	−0.031	−0.004	0.010	*−0.001		
Sex	0.279	0.174	0.366	−1.162	**−2.715	***−0.041	**−0.049	
Trials	−0.013	−0.015	−0.010	−0.009		0.001		
System	0.428	0.840	0.738	0.090	*1.801			
Age*Group	**0.055	*0.050	**0.063	~ 0.041		**0.001		
Age*System	*−0.055	*−0.076	*−0.075	***−0.140	***−0.106			
Age*Trials								
Sex*Group	**−3.704	*−3.645	**−4.390	**−4.423				
Sex*System	**3.808	*3.633	1.429	~ 2.615				
Sex*Trials						−0.000		
Group*System	1.839	2.043	*4.320	~ 3.176				
Group*Trials	**−0.084	*−0.083	*−0.078	**−0.084				
System*Trials	*0.104	~ 0.083	~ 0.085					
**Omnibus F**	F(12, 61) = 11.6	F(12, 61) = 9.11	F(12, 61) = 8.34	F(11, 62) = 9.23	F(4, 69) = 9.40	F(6, 67) = 6.88	F(3, 70) = 8.84	F(1, 73) = 105
**Model** ***p*-value**	0.000	0.000	0.000	0.000	0.000	0.000	0.000	0.000
**Mean**	52.4	51.2	47.5	39.9	24.8	0.046	0.093	0.126
	0.695	0.642	0.621	0.621	0.353	0.381	0.275	0.000
**Univariable: Stim 1**	**−1.7 (−4.3, 0.1)**	−1.1 (−4.0, 1.4)	−1.1 (−3.3, 1.1)	−0.1 (−2.1, 2.0)	−0.2 (−3.1, 2.3)	*−0.0 (−0.1, −0.0)	~**−0.1 (−0.1, 0.0)**	−0.0 (−0.1, 0.0)
**Univariable: Stim 2**	**−1.7 (−4.0, 0.4)**	−1.3 (−4.0, 1.1)	−1.0 (−3.5, 1.2)	0.0 (−2.1, 2.2)	−0.5 (−3.1, 2.3)	−0.0 (−0.1, 0.0)	0.0 (−0.0, 0.0)	−0.0 (−0.1, 0.1)

Note: Table of parameter estimates. Each column represents a group LASSO model of an EEG outcome variable. Parameter estimates less than 0.001 were shortened to 0.000 to save space. ~*p* <.10, *p <.05, **p <.01, ***p <.001. Variables with no symbol were retained by group LASSO but not significant in the final model. A non-significant model means that independent variables did not improve model fit over an intercept-only model. Only variables chosen by group LASSO are presented. Reference groups are as follows—Stimulus: Stimulus 1, Sex: Male, Group: TD, System: EGI. ITC, intertrial phase coherence; Stim, stimulus; TD, typically developing; PMS, Phelan McDermid Syndrome. The univariable rows indicate the median difference and 95% confidence interval between PMS and TD for each EEG outcome variable to either the first or second auditory stimulus (for additional details on the univariable analyses, see Supplemental Table 2). For these univariable results, negative values are lower for PMS vs. TD, and positive values are higher for PMS vs. TD. The bold highlights potentially clinically relevant cells in which *p* <.25. ~*p* <.10, *p <.05, **p <.01, ***p <.001

**Table 6:** Whole-Head Time-Frequency results: between PMS and TD

Variable	Delta (µV^2^)	Theta (µV^2^)	Alpha (µV^2^)	Beta (µV^2^)	Gamma (µV^2^)	Beta-gamma (ITC)	Alpha-beta (ITC)	Delta-theta (ITC)
Group	0.218	0.860	0.887	**1.522	0.272			
Age	***−0.071	***−0.062	**−0.033	*−0.021	*0.020			
Sex	***1.887	***2.823	***2.572	**1.578	−0.603		**−0.017	
Trials	−0.005	−0.000	0.010	0.009	0.013			
System	***2.119	***2.243	***1.925	***1.804	~ 1.001			
Age*Group	**0.042	*0.028	*0.027	0.017				
Age*Gender			*−0.021					
Age*System			***−0.055	***−0.054	***−0.083			
Sex*Group		*−1.992	*−2.121	***−2.809				
Group*Trials	**−0.051	**−0.042	***−0.065	***−0.060	***−0.080			
**Omnibus F**	F(7, 66) = 20.52	F(8, 65) = 21.51	F(10, 63) = 17.29	F(9, 64) = 14.65	F(7, 66) = 5.54	F(1, 73) = 17.93	F(1, 72) = 9.85	F(1, 73) = 33.68
**Model** ***p*-value**	0.000	0.000	0.000	0.000	0.000	0.000	0.003	0.000
**Mean**	45.2	44.6	42.8	37.5	28.4	0.012	0.017	0.024
***R***	0.685	0.726	0.733	0.673	0.370	0.000	0.120	0.000
**Univariable: Stim 1**	**−1.3 (−3.2, 0.6)**	~**−1.5 (−3.5, 0.2)**	~**−1.5 (−3.0, 0.4)**	−0.5 (−1.8, 1.0)	1.0 (−0.8, 2.5)	~**−0.0 (−0.0, 0.0)**	~**−0.0 (−0.0, 0.0)**	−0.0 (−0.0, 0.0)
**Univariable: Stim 2**	−0.8 (−2.8, 1.2)	**−1.1 (−3.0, 0.7)**	**−1.3 (−2.9, 0.5)**	−0.4 (−1.7, 1.3)	0.9 (−0.9, 2.2)	0.0 (−0.0, 0.0)	−0.0 (−0.0, 0.0)	−0.0 (−0.0, 0.0)

Note: Table of parameter estimates. Each column represents a group LASSO model of an EEG outcome variable. Parameter estimates less than 0.001 were shortened to 0.000 to save space. ~*p* <.10, *p <.05, **p <.01, ***p <.001. Variables with no symbol were retained by group LASSO but not significant in the final model. A non-significant model means that independent variables did not improve model fit over an intercept-only model. Only variables chosen by group LASSO are presented. Reference groups are as follows—Stimulus: Stimulus 1, Sex: Male, Group: TD, System: EGI. ITC, intertrial phase coherence; Stim, stimulus; TD, typically developing; PMS, Phelan McDermid Syndrome. The univariable rows indicate the median difference and 95% confidence interval between PMS and TD for each EEG outcome variable to either the first or second auditory stimulus (for additional details on the univariable analyses, see Supplemental Table 2). For these univariable results, negative values are lower for PMS vs. TD, and positive values are higher for PMS vs. TD. The bold highlights potentially clinically relevant cells in which *p* <.25. ~*p* <.10, *p <.05, **p <.01, ***p <.001

### Additional analysis: stimulus, age, sex, and deletion size

#### Stimulus

Across PMS and TD groups, stimulus was significantly associated with P50 and N1 latencies and P2 amplitudes (Table 4; Fig. 1), in which latencies were longer and amplitudes were weaker for stimulus 2 than for stimulus 1—a normal gating effect. In PMS only analyses, fewer components included stimulus as an important model feature—only for models of P50 latency and N1 amplitude (Table 7). This could indicate a moderately dampened gating response in PMS, particularly for P2 amplitudes, which is also suggested by Fig. 1a, b. In the frequency domain, alpha-beta ITC had a typical gating effect for PMS vs. TD and PMS only analyses, while power and other phase-locking variables were either more stable across stimuli or did not include stimulus effects after model selection (Tables 5 and 8).

**Table 7:** Frontal ERP results: PMS only

Variable	P50A (µV)	P50L (ms)	N1A (µV)	N1L (ms)	P2A (µV)	P2L (ms)	N2A (µV)	N2L (ms)
Deletion Size	*−0.275	3.209	0.043					2.471
Stim		*27.51	*0.478					
Age		−0.190	0.001			~−0.303	0.003	
Sex	−0.274	1.588						
Trials		0.173	−0.002		−0.006		0.005	0.236
System		3.458						
DS*Age			~ 0.005					
DS*Stim		~−7.496						
DS*Sex	*0.378							
DS*Trials			*0.004					*−0.363
DS*System		3.383						
Stim*Trials		−0.060						
Stim*System		*−44.20						
Age*Sex		0.262						
Age*Trials							*0.000	
Age*System		−0.148						
**Omnibus F**	F(3, 38) = 3.28	F(12, 29) = 1.93	F(6, 35) = 2.63	F(1, 41) = 566	F(1, 40) = 1.48	F(1, 40) = 3.16	F(3, 38) = 2.50	F(3, 38) = 2.51
**Model** ***p*-value**	0.031	0.073	0.033	0.000	0.231	0.083	0.074	0.073
**Mean**	1.40	93.1	− 0.774	161	0.854	219	− 0.998	295
	0.206	0.444	0.311	0.000	0.036	0.073	0.165	0.166

Note: Table of parameter estimates. Each column represents a group LASSO model of an EEG outcome variable. Parameter estimates less than 0.001 were shortened to 0.000 to save space. ~*p* <.10, *p <.05, **p <.01, ***p <.001. Variables with no symbol were retained by group LASSO but not significant in the final model. A non-significant model means that independent variables did not improve model fit over an intercept-only model. Only variables chosen by group LASSO are presented. Reference groups are as follows—Stimulus: Stimulus 1, Sex: Male, Group: TD, System: EGI. DS, deletion size; A, amplitude; L, latency; Stim, stimulus; PMS, Phelan McDermid Syndrome

**Table 8:** Frontal Time-Frequency results: PMS only

Variable	Delta (µV^2^)	Theta (µV^2^)	Alpha (µV^2^)	Beta (µV^2^)	Gamma (µV^2^)	Beta-Gamma (ITC)	Alpha-Beta (ITC)	Delta-Theta (ITC)
Deletion Size	~−0.719	−0.699	−0.610		*−0.857			
Stim						−0.003	*−0.038	
Age	~−0.042	*−0.055	−0.016		*0.046	0.000	−0.000	
Sex	*−2.563	*−2.660	*−3.649	***−6.044	*−2.979	~−0.015	*−0.050	
Trials	~−0.057	−0.033	−0.051	−0.031	***−0.130	***0.002	***0.002	~ 0.001
System	~ 2.619	~ 3.575	*4.597	1.954	**3.519	0.022	~−0.072	
DS*Stim							0.000	
DS*Age	**−0.036	**−0.044	~−0.027					
DS*System					0.874			
Stim*Trials						~−0.001		
Age*Sex						*−0.000		
Age*System	−0.041				***−0.227		**0.002	
Age*Trials						***0.000	0.000	
Sex*System	2.976	2.217	1.821	4.152			*0.042	
Sex*Trials						−0.000		
System*Trials	0.041	0.038	0.080			**−0.002		
**Omnibus F**	F(9, 32) = 9.67	F(8, 33) = 8.18	F(8, 33) = 5.53	F(4, 37) = 6.30	F(7, 34) = 7.79	F(10, 31) = 6.40	F(8, 33) = 5.85	F(1, 40) = 3.32
**Model** ***p*-value**	0.000	0.000	0.000	0.001	0.000	0.000	0.000	0.076
**Mean**	51.5	50.5	46.8	39.6	24.3	0.030	0.075	0.115
***R *** ^2^	0.731	0.665	0.573	0.405	0.616	0.674	0.587	0.077

Note: Table of parameter estimates. Each column represents a group LASSO model of an EEG outcome variable. Parameter estimates less than 0.001 were shortened to 0.000 to save space. ~*p* <.10, *p <.05, **p <.01, ***p <.001. Variables with no symbol were retained by group LASSO but not significant in the final model. A non-significant model means that independent variables did not improve model fit over an intercept-only model. Only variables chosen by group LASSO are presented. Reference groups are as follows—Stimulus: Stimulus 1, Sex: Male, Group: TD, System: EGI. ITC, intertrial phase coherence; DS, deletion size; Stim, stimulus; PMS, Phelan McDermid Syndrome

#### Deletion size

Larger deletion sizes within PMS were associated with decreased frontal P50 amplitudes, and more-so for males (Table 7), suggesting impaired cortical registration of the auditory stimuli or poor attenuation of repeated stimuli among the largest deletion sizes. In frequency space, individuals with larger deletion sizes also had less gamma power (Table 8). Given the abnormal gating and auditory processing effects described from ERP analyses, low gamma power may be associated with additional sensory hyposensitivity for individuals with larger deletion sizes. Additionally, a deletion size by age interaction suggested that delta and theta power decreased most by age for individuals with larger deletions (Table 8; see Supplemental Fig. 3 for an example visualization), similar to whole-head delta, theta, and alpha power (Table 9). These findings could suggest that the severity of early auditory processing impairment in PMS is influenced by additional genetic material in exonal regions near *SHANK3* and that the impact of larger deletion sizes is sensitive to age.

**Table 9:** Whole-Head Time-Frequency results: PMS only

Group	Delta (µV^2^)	Theta (µV^2^)	Alpha (µV^2^)	Beta (µV^2^)	Gamma (µV^2^)	Beta-Gamma (ITC)	Alpha-Beta (ITC)	Delta-Theta (ITC)
Deletion Size	~−0.353	*−0.420	*−0.382		−0.151			
Age	***−0.052	***−0.059	***−0.048	0.003		*0.000		**0.000
Sex	*1.456	*1.121		**−2.016	−1.561	0.001		
Trials	***−0.050	***−0.039	*−0.033	***−0.075	−0.030	0.000		
System	1.071	~ 1.005	0.954	−0.162	*1.923			
DS*Age	***−0.023	***−0.025	***−0.024					
Stim*Trials						~−0.001		
Age*Sex						*−0.000		
Age*System			−0.028	***−0.071				
Age*Trials	~−0.001	*−0.001	*−0.001	*−0.001		**0.000		
Sex*System				1.760				
Sex*Trials				~ 0.039				
**Omnibus F**	F(7, 34) = 18.54	F(7, 34) = 24.83	F(7, 34) = 18.16	F(8, 33) = 9.73	F(4, 37) = 2.40	F(7, 34) = 3.04	F(1, 41) = 19.36	F(1, 40) = 8.55
**Model** ***p*-value**	0.000	0.000	0.000	0.000	0.068	0.014	0.000	0.006
**Mean**	44.8	44.1	42.3	37.3	28.5	0.011	0.012	0.019
	0.792	0.836	0.789	0.702	0.206	0.385	0.000	0.176

Note: Table of parameter estimates. Each column represents a group LASSO model of an EEG outcome variable. Parameter estimates less than 0.001 were shortened to 0.000 to save space. ~*p* <.10, *p <.05, **p <.01, ***p <.001. Variables with no symbol were retained by group LASSO but not significant in the final model. A non-significant model means that independent variables did not improve model fit over an intercept-only model. Only variables chosen by group LASSO are presented. Reference groups are as follows—Stimulus: Stimulus 1, Sex: Male, Group: TD, System: EGI. ITC, intertrial phase coherence; DS, deletion size; Stim, stimulus; PMS, Phelan McDermid Syndrome

#### Age

No unique age effects were found for PMS in frontal ERP analyses. However, older individuals were found to have lower delta power and beta-gamma ITC across PMS and TD (Table 5), while within-PMS data had lower theta and higher gamma power—potentially suggesting unique involvement of gamma or theta in developmental differences between PMS and TD (Table 8). Males and females with PMS also differed by age in frontal and whole-head beta-gamma ITC such that older females had less high-frequency phase locking than older males, which may be driving the age-related discrepancy between PMS and TD beta-gamma ITC (see Supplemental Fig. 4 for an example visualization). Whole-head delta-theta ITC similarly increased in PMS with age (Table 9)—ITC patterns that were absent in PMS vs. TD analysis (Table 6).

#### Sex

Females across PMS and TD had lower frontal P2 amplitudes than males (Table 4). This effect did not appear in the PMS-only analyses (Table 7), which may indicate that the effect is largely driven by the TD group (Table 4). In the frequency domain, males across PMS and TD had higher frontal gamma power and alpha-beta and beta-gamma ITC than females (Table 5); males within PMS had higher delta through gamma power and alpha-beta ITC suggesting a unique sex effect for delta through beta power in PMS (Table 8). These sweeping differences in frontal power are likely explained by females with PMS having abnormally low power compared to TD or males with PMS, as described previously.

#### Frontal vs. Whole-head

**Fig. 3:**
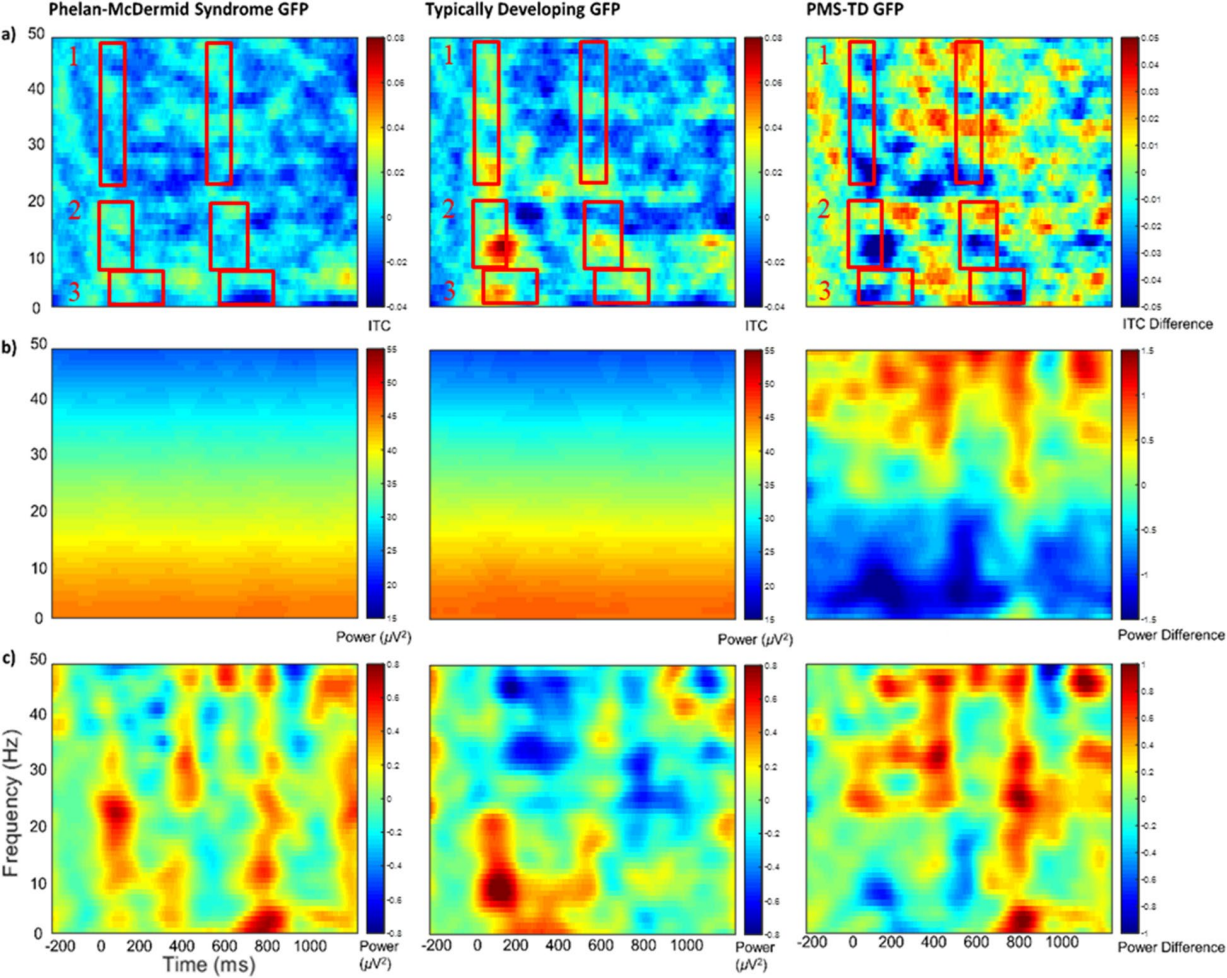
Whole-Head Time-Frequency Analysis. (a) Inter-trial phase coherence (unitless). ITC values were averaged across the indicated time and frequency ranges. (b) Single-trial power as event-related spectral perturbation (µV^2^). (c) Baseline-corrected single-trial power (µV^2^). Warmer colors indicate higher phase locking and power values, while cooler colors indicate lower values. GFP, global field power; PMS, Phelan-McDermid Syndrome; TD, Typically Developing

The global or whole-head approach demonstrated several unique effects for PMS. For instance, group differences in N1 and P2 amplitudes were more apparent in the whole-head analyses, likely due to the variations in N1 and P2 topographies across development or between groups that were incompletely captured by the frontal electrode subset (Table 10; Supplemental Fig. 5). Whole-head analyses also showed that individuals with PMS dropped an additional 0.012µV in N1 GFP for every month increase in age compared to TD, suggesting greater developmental divergence between groups in auditory N1 feature processing. In the frequency domain, a novel and unexpected group difference indicated higher whole-head beta power for PMS (Table 6; Fig. 3b, c), while age-related group differences in the frequency domain were similar for frontal and whole-head approaches.

**Table 10:** Whole-Head ERP results: between PMS and TD

Variable	P50 GFP (µV)	P50L (ms)	N1 GFP (µV)	N1L (ms)	P2 GFP (µV)	P2L (ms)	N2 GFP (µV)	N2L (ms)
Group		***17.47	−0.184		***−0.449	***41.25		
Stim	*−0.380	***30.08	***−0.878		***−0.492			
Age	−0.003		0.006				***−0.005	
Sex	*−0.360		**−0.575		**−0.341			
Trials	0.005		−0.004		*−0.006			
System	0.271	3.911						
Group*Age			*−0.012					
Stim*Sex			~ 0.512					
Stim*System		***−41.78						
Age*System	*−0.015							
Age*Trials								
Sex*Trials	*−0.013							
Trials*System								
**Omnibus F**	F(7, 66) = 5.75	F(4, 69) = 13.26	F(7, 59) = 6.79	F(1, 66) = 864	F(4, 67) = 11.97	F(1, 70) = 19.81	F(1, 70) = 12.30	F(1, 71) = 2952
**Model** ***p*-value**	0.000	0.000	0.000	0.000	0.000	0.000	0.001	0.000
**Mean**	1.50	86.9	1.18	147	1.27	199	1.13	285
**R ** ^2^	0.379	0.435	0.446	0.000	0.417	0.220	0.149	0.000
**Univariable: Stim 1**	*−0.4 (−0.9, −0.0)	**10 (−4, 22)**	~**−0.5 (−1.0, 0.1)**	**−21 (−50, 4)**	*−0.6 (−1.0, −0.1)	**−18 (−42, 4)**	~**−0.4 (−0.8, 0.0)**	4 (−22, 36)
**Univariable: Stim 2**	−0.1 (−0.4, 0.1)	5 (−10, 26)	−0.1 (−0.4, 0.2)	−4 (−32, 18)	−0.2 (−0.5, 0.1)	−2 (−44, 24)	**−0.2 (−0.5, 0.0)**	14 (−18, 50)

Note: Table of parameter estimates. Each column represents a group LASSO model of an EEG outcome variable. Parameter estimates less than 0.001 were shortened to 0.000 to save space. ~*p* <.10, *p <.05, **p <.01, ***p <.001. Variables with no symbol were retained by group LASSO but not significant in the final model. A non-significant model means that independent variables did not improve model fit over an intercept-only model. Only variables chosen by group LASSO are presented. Reference groups are as follows—Stimulus: Stimulus 1, Sex: Male, Group: TD, System: EGI. GFP, global field power; L, latency; Stim, stimulus; TD, typically developing; PMS, Phelan McDermid Syndrome. The univariable rows indicate the median difference and 95% confidence interval between PMS and TD for each EEG outcome variable to either the first or second auditory stimulus (for additional details on the univariable analyses, see Supplemental Table 2). For these univariable results, negative values are lower for PMS vs. TD, and positive values are higher for PMS vs. TD. The bold highlights potentially clinically relevant cells in which *p* <.25. ~*p* <.10, *p <.05, **p <.01, ***p <.001

Whole-head analyses had more consistent gating effects as well—with expected decreases in P50, N1, and P2 GFP and P50 latency for stimulus 2 (Table 10; Fig. 1). Like frontal analyses, PMS models had fewer selected gating effects—reduced to N1 and P2 GFP and N1 latency (Table 11). Within PMS, larger deletion sizes were associated with shorter P50 and N1 latencies for stimulus 2 vs. 1 (Table 11), suggesting larger global delays and a more abnormal early auditory gating responses for the largest deletion sizes. Theta and alpha power also differed by deletion size (Table 9), unlike the effect of gamma in frontal analyses, potentially suggesting that deletion-size-related auditory impairments in PMS are related to whole-head disruptions in information integration (theta) and sensory processing or selective auditory inhibition (alpha).

**Table 11:** Whole-Head ERP results: PMS only

Group	P50 GFP (µV)	P50L (ms)	N1 GFP (µV)	N1L (ms)	P2 GFP (µV)	P2L (ms)	N2 GFP (µV)	N2L (ms)
Deletion Size		**5.818		**9.899			~−0.217	
Stim		~ 12.38	**−0.568	***51.10	*−0.423			
Age	*−0.005		−0.003	0.175			~−0.004	
Sex			**−0.503	**38.04			0.123	
Trials			~−0.010	−0.294	*−0.008		*−0.009	
System		**−19.86	−0.074	***70.54				
DS*Stim		**−10.27		***−21.70				
DS*Sex							*0.321	
DS*System				**13.54				
Stim*Age				*−0.515				
Stim*Sex				*−37.86				
Stim*System				***−75.93				
Age*System			~−0.010					
Sex*Trials				*1.022				
Sex*System				***−83.91				
Trials*System			~ 0.015	**1.348				
**Omnibus F**	F(1, 40) = 5.33	F(4, 37) = 5.79	F(6, 33) = 5.12	F(14, 25) = 7.09	F(2, 38) = 5.83	F(1, 40) = 860	F(5, 34) = 4.27	F(1, 39) = 1242
**Model** ***p*-value**	0.026	0.001	0.001	0.000	0.006	0.000	0.004	0.000
**Mean**	1.41	94.0	1.10	160	1.05	217	0.995	293
**R**	0.118	0.385	0.482	0.799	0.235	0.000	0.386	0.000

Note: Table of parameter estimates. Each column represents a group LASSO model of an EEG outcome variable. Parameter estimates less than 0.001 were shortened to 0.000 to save space. ~*p* <.10, *p <.05, **p <.01, ***p <.001. Variables with no symbol were retained by group LASSO but not significant in the final model. A non-significant model means that independent variables did not improve model fit over an intercept-only model. Only variables chosen by group LASSO are presented. Reference groups are as follows—Stimulus: Stimulus 1, Sex: Male, Group: TD, System: EGI. DS, deletion size; GFP, global field power; L, latency; Stim, stimulus; PMS, Phelan McDermid Syndrome

Age and sex effects occurred more often in whole-head analyses than frontal analyses. Across PMS and TD, frontal N2 amplitudes increased as age increased (Table 4), while whole-head N2 GFP appeared to decrease (Table 10)—suggesting an increased frontal focality of N2 activity with age. Within PMS, P50 GFP decreased with age, highlighting a unique within-PMS effect (Table 11). Additionally, for every unit increase in age, whole-head PMS N1 latencies decreased more for stimulus 2 than 1, which differed from TD findings and may suggest an increasingly abnormal or blunted gating response with age in PMS. For frequency-based measures across PMS and TD, there was lower delta through beta power but higher gamma power per unit increase in age (Table 6). Within PMS effects were similar, although age did not have an apparent effect on whole-head beta or gamma power, which may indicate that (1) differences between PMS and TD in whole-head beta are not driven by age and (2) that developmental effects of gamma power on PMS are frontally localized.

By sex, females had weaker P50, N1, and P2 GFP peaks than males, which is a more extensive finding than the frontal difference in P2 amplitudes (Table 10). However, this effect may be driven primarily by TD; within PMS, only whole-head N1 GFP and N1 latency differed by sex, in which females with PMS had weaker amplitudes and longer latencies. Interestingly, in the frequency domain, whole-head delta, theta, alpha, and beta power were significantly *larger* for females across PMS and TD, opposite of frontal findings, although alpha-beta ITC was still reduced in females (Table 6). This may indicate a cohort-wide upregulation of global processing and down-regulation in frontally localized activity for females vs. males. Within the PMS group, whole-head delta and theta power were similarly large for females (Table 9). However, PMS-only beta power was significantly higher in males (Table 9) and thus may be driving primary differences between PMS and TD in beta power.

#### EEG system and trial count

These variables were included as potential confounding factors. Descriptions of system and trial count effects can be found in Appendix 1 of supplemental materials and in Tables 4–11.

#### Supplemental univariable analyses

Both full and age-restricted sub-analyses of median PMS vs. TD differences in EEG auditory processing outcomes by auditory stimulus demonstrate a consistent impact of age on whole-head metrics, particularly for GFP and low frequency (delta, theta, alpha) power (Supplementary Table 2). This parallels multivariable whole-head power findings that present significant effects of age (Table 6). Whole-head GFP models did not have significant effects of age but did include this variable as an important covariate (except for P2 GFP, Table 10). Frontal beta-gamma ITC remained significant in the full and age-restricted analyses and was significant in group LASSO models (Table 5), suggesting a robust finding of reduced beta-gamma ITC in PMS.

Further, frontal N1 latency and whole-head P50 and P2 GFP had notable decreases in significance after age-restricting the univariable analyses, though frontal alpha-beta ITC, whole-head P2 latency, and whole-head delta-theta ITC had notable increases in significance in this analysis. Other variables, such as frontal P2 latency and various whole-head metrics (P50 and N1 latency, N1 and N2 GFP, theta, alpha, beta-gamma ITC, and alpha-beta ITC), changed in more subtle ways with age-restriction. Importantly, these variables appear sensitive to age-related changes and suggest either potential underpowered analyses or age-related effects to be explored in future studies.

## Discussion

Results showed a variety of differences in group, deletion size, age, and sex for PMS. Major group differences are discussed first, followed by specific stimulus-gating effects and effects of other group characteristics.

### PMS vs. TD

In line with expectations, individuals with PMS had delayed or low amplitude P50, N1, and P2 components across frontal and whole-head analyses as well as weak phase-locking in the alpha to gamma ranges, indicating widespread impairment in auditory processing of stimulus properties in PMS. Given theorized mechanisms of these ERP and ITC features, the extent of these auditory impairments could include abnormalities in cortical registration or recognition of auditory tones and delayed identification or filtering of repetitive stimuli. Thus, it is possible that the identified impairments are partially responsible for difficulty with sensory responsiveness and speech development in PMS [12]. Indeed, some of these EEG measures were correlated with nonverbal IQ and Vineland Communication Score, including low frequency power and phase-locking (Table 2). Additional age-related delta, theta, alpha power, and frontal beta-gamma ITC differences suggested that TD and PMS individuals are undergoing different developmental trajectories. Thus, it may be that young individuals with PMS enter late critical periods with abnormally low power and differential phase-locking patterns, which may be reflective of an excitatory/inhibitory imbalance that is later compensated for in adulthood. Specifically, low delta power during development may lead to impaired inhibitory control later in life [25], while low theta and alpha power may lead to impaired thalamocortical modulation [26], both of which could contribute to impairments in cortical registration or recognition of auditory tones in young individuals with PMS. Additionally, weak frontal beta-gamma phase-locking could suggest a disruption in information processing and coordination across brain regions during development [28], with potential downstream effects on higher-order processes such as language processing and speech development in PMS.

Contrary to hypotheses, PMS did not have reduced power in direct multivariable PMS vs. TD comparisons; in fact, PMS had higher whole-head beta power than TD—and males with PMS likely drove this difference. The general lack of power differences between PMS and TD may also be driven by males with PMS, who otherwise had relatively normal power levels; whereas females with PMS had a stark decrease in power across all frequencies compared to both males with PMS and TD. Sex-based differences may be influenced by differences in deletion size as, described in the results, or by differences in medication use. Among 16 participants with reported medication data, 9 females vs. 2 males were recorded as receiving some form of psychoactive medication (see Supplementary Table 1 for medication details). Considering the significant sex difference in deletion size and potential power differences due to medication, it is unclear whether the sex-based difference in PMS is clinically meaningful. Interestingly, increases in delta, theta, and alpha power in PMS were associated with increases in autistic features as well as decreases in nonverbal IQ and communication skills, suggesting a complex interplay between power reductions, sex, and clinical outcome that should be explored in future studies.

### Stimulus

No group by stimulus interactions between PMS and TD were chosen by group LASSO, which suggests that gating interactions did not improve adjusted R^2^ enough to be considered an important model feature. The lack of gating differences between the entire PMS and TD cohort complements recent work by Isenstein et al., which showed comparable auditory gating between individuals with PMS and neurotypical controls [54]. The present study expands upon these findings by exploring gating interactions within PMS. An age-by-stimulus interaction for whole-head N1 latency showed less of a discrepancy between stimulus 1 and 2 latencies for older individuals, which may indicate a dampened gating response with age in PMS. Individuals with larger deletion sizes had similarly abnormal gating responses for whole-head P50 and N1 latencies, which may indicate a deletion-size dependent gating abnormality for P50 and N1 latency in PMS. Results from [54] demonstrated a similar association between deletion size and N1 gating amplitudes using univariate analyses. Further study of gating in PMS is necessary to delineate potential age or deletion size dependent changes of sensory gating responses. Despite inconclusive gating differences, findings for EEG amplitude, latency, power, and deletion size fit sensory hyposensitivity descriptions of PMS.

### Deletion size

Larger deletion sizes were expected to be associated with greater auditory processing abnormalities (i.e., lower amplitude ERPs, longer latencies, higher gamma and lower power otherwise, reduced phase-locking, and less effective gating). Overall, individuals with larger deletion sizes had atypical frontal P50 amplitudes and whole-head P50 and N1 latencies. Since the P50 is related to early stimulus identification and filtering out redundant information, and the N1 is related to pattern and detail recognition in auditory gating, individuals with large *SHANK3* deletions may have more difficulties identifying sounds in context than counterparts with smaller deletions. This implies a phenotypic difference in auditory response according to deletion size, in which larger deletion sizes are associated with increased auditory processing abnormalities for P50 and N1. This fits with the idea that larger deletion sizes affect a greater range of genes, which may have additive impact on the severity and range of PMS symptomology [1, 55]. For instance, *IB2* (or *MAPK8IP2*) and *PARVB* are two morphogenetic genes proximal to *SHANK3*. *IB2* is concentrated in the postsynaptic density and affects synaptic transmission and dendritic morphology; an *IB2* knockout mouse model demonstrated poor sensorimotor processing and reduced social interaction compared to wild-type mice, which may similarly compound sensory and social deficits in human PMS with *IB2* deletion [56]. *PARVB* is a scaffolding protein in the postsynaptic density, like *SHANK3*, and is associated with ASD, absent speech, and sensory sensitivity, which may similarly contribute to hyposensitivity among individuals with PMS and *PARVB* deletion [55].

In frequency space, larger deletion sizes were associated with decreased frontal gamma. Interestingly, gamma power is consistently found to be abnormal in Fragile X Syndrome and ASD [9, 57], and is abnormally modulated by alpha phase in PMS and ASD [10, 58], which may highlight the usefulness of this frequency band for characterizing PMS phenotypes. In fact, it is possible that ERP, power, and gating-specific measures represent different mechanisms that could benefit understanding of differences between neurodevelopmental disorders with different combinations of sensory processing abnormalities. For instance, poor gating and high or low gamma power and ERP amplitudes (e.g., N1 [9]) may reflect sensory hyper-responsiveness or hypo-responsiveness, respectively, and may help distinguish subtypes of idiopathic autism.

Deletion size also altered effects of age on frontal delta and theta power, suggesting differential effects of deletion size across development—potentially though neural reorganization in response to sensory processing impairment. In short, larger deletions had greater auditory processing impairment than smaller deletions in this sample. Thus, deletion sizes may account for the wide variation in speech and language abilities among PMS patients through extended deletion of neighboring genes that contribute to additional auditory processing impairments [1, 55]. Future studies should further investigate the possibility of developmentally regulated changes in the effect of deletion size on phenotypes in PMS.

### Age

In line with developmental expectations, younger individuals with PMS typically had higher whole-head P50 amplitudes, elevated low-mid frequency frontal and whole-head oscillations, less frontal gamma, and less phase locking compared to older PMS participants. Given that auditory processing abnormalities may exist in younger PMS individuals and that discrepancies between PMS and TD in auditory processing may grow with age, early childhood is a logical target for sensory development therapies. Researchers have already determined that patients with PMS benefit from early intervention programs along with sport and exercise therapy to strengthen their muscles and regular, intense therapies for improving communication [59]. Additionally, P50 amplitude, gamma or theta power, and delta-theta ITC measures may be effective biomarkers for investigating the efficacy of therapies aimed at improving underlying sensory processing abnormalities in young individuals with PMS. Since physiological changes are often detectable before behavioral consequences, neurophysiological measures are important for early detection [60].

### Sex

The findings showed sex differences in EEG outcomes within PMS as well as between genetic group (PMS vs. TD). Females were hypothesized to have higher amplitude ERPs than males, which was opposite of what was generally found in frontal and whole-head analyses both in the overall cohort and in PMS only analyses. Several sex-based group differences are discussed in the section on PMS vs. TD. As mentioned previously, given significant differences in deletion size by sex, it is unclear if these sex-related differences are clinically meaningful for PMS. Potential differential effects of sex on auditory processing in PMS should be further investigated.

### Frontal vs. Whole-Head

Due to predicted developmental delays in topographic distribution of auditory sources in PMS, we pursued both regional (frontal) and global (whole-head) analyses. Frontal analyses averaged over electrodes relevant to mature auditory gating patterns were predicted to identify larger between-group differences in neural activity than whole-head analyses. Altogether, the frontal approach captured a different neurophysiological response between PMS and TD groups than the whole-head approach. For instance, frontal analyses better captured general differences in ITC aside from age-related ITC effects within PMS. This may suggest that auditory task-related phase-locking was generally localized to the frontal lobe for TD but was more varied in PMS with development. Frontal analyses also showed stronger differences in gamma power, while whole-head analyses were more sensitive to other power-related differences and differences by age, particularly in the frequency domain. These whole-head effects could suggest that developmental differences consisted of more widespread alterations of neural networks rather than local processing or that participants had widely varied topographies across developmental stages that were better captured with a whole-head approach. Thus, the choice of one approach versus the other should be directed according to hypotheses regarding global dysfunction of neural networks versus localized impairment and should be carefully weighed when working with a developmentally varied sample.

### Limitations

Several limitations must be acknowledged. (1) The age range for the PMS group (8-18.6 years) was wider than for the TD group (8.2–15.3 years). This suggests a need for cautious interpretation of age-related effects, given the potential confounding of age and overall group differences. While we include supplemental univariable analyses in the full vs. age 8–12 restricted sample to delineate the potential impact of age discrepancies on group findings, a larger study is warranted to investigate auditory ERP differences between age-matched PMS and TD at different developmental stages. (2) Importantly, our data has relatively low statistical power for subgroup comparisons, such as by PMS and TD, and some participants lacked a complete set of clinical measures. General sample size issues are difficult to overcome for studies of PMS, given that this is a rare genetic condition. Analyses were also restricted to participants over age 8 due to the topographical variation among younger participants. Only 2 subjects under age 8 were controls; thus, all subjects < 8 were removed to avoid biases due to the non-random, highly imbalanced set of subjects with developmentally-varied EEG topographies. Although this is one of the largest EEG studies of PMS to date, the exploratory nature of the hypotheses is purely intended to guide new studies of PMS. Future research may aim for a sample size of at least 5% of the population, around 100 individuals with PMS, to perform finite population correction. This correction reduces the required sample size for a small population and adjusts variance and mean estimates to account only for the proportion of the population absent from the sample [61, 62].

3) Our finding that individuals with PMS had significantly delayed latencies compared to TD falls in line with clinical evidence [21, 32] and animal models [23, 39] of PMS that suggest hyposensitivity to sensory stimuli. Our univariable results were similar to recent auditory EEG findings from Isenstein et al., which suggest relatively preserved ERP amplitudes and habituation responses between PMS and TD and potentially clinically relevant within-PMS associations between N1 and P2 magnitudes and sensory processing deficits [44]. In this study, these within-PMS correlations were between N1, P2 GFP and SSP Total scores. However, there was a discrepancy in latency findings between Isenstein et al. and our multivariable models, which may be due to differences in sample selection or statistical approaches. The multivariable group LASSO models in the current study were designed to account for important group differences by stimulus, age, sex, trial count, and EEG system, which have demonstrated impact on EEG outcomes. This idea is generally supported by the lack of robust univariable latency findings in this study that emphasize the importance of accounting for factors such as age.

Notably, we chose the data-driven group LASSO approach as our primary reported analysis due to the abundance of variables that had strong theoretical foundations for potential inclusion in our models. Our limited sample size in this rare cohort precluded inclusion of all variables of interest. However, the group LASSO approach offered a powerful means of selecting the most optimally relevant, practically important features for each EEG outcome in the specific context of our variable list. If a group LASSO model did not include, e.g., age as a covariate, then this indicated that the EEG outcome variable was not significantly influenced by age in the presence of the other group LASSO-selected variables, or that the unique contribution of age to prediction was minimal overall. Group LASSO selection included variables only when they were important contributors to the overall variance in the EEG outcome explained by the model. Thus, we believe that the primary reported multivariable group LASSO results are crucial for this cohort, given that they account for variability explained by covariates age, sex, trial count, EEG system, and study group (or deletion size) in this exceedingly rare sample. Importantly, the significant PMS vs. TD effects that group LASSO identified align with the overall data patterns observed in our descriptive Table 3 and align with existing literature. Thus, this complex analysis provides important context for future studies, and our supplemental univariable analyses provide complementary information regarding general patterns of group differences in the full vs. age 8-12-restricted samples.

## Conclusions

In conclusion, PMS individuals experienced a variety of ERP and power-related auditory processing abnormalities compared to typically developing (TD) individuals, even after controlling for demographic variables. Individuals with PMS had delayed ERPs and weaker power and phase-locking to auditory stimuli with noted developmental divergences from TD. Additionally, larger deletions demonstrated unique auditory impairments, which could indicate particular difficulty filtering sensory information and highlights an important topic for future research. Thus, despite the complex interrelationships between demographic, clinical, and EEG factors, the univariable and multivariable findings emphasize the importance of factors such as deletion size, age, and sex for understanding neurophysiological outcomes in this population as well as the utility of EEG data as biomarkers for sensory processing in PMS. This includes potential use in early identification or individualized therapies for PMS patients, as well as biomarkers in clinical trials to track the efficacy of treatments, or as a tool to further investigate neurophysiological mechanisms of PMS impairments.

## Supplementary Information

Below is the link to the electronic supplementary material.

Supplementary Material 1

## Data Availability

The datasets used and/or analyzed in this study are available from the corresponding author upon reasonable request.
