# Supplementary material for: Phenotypic variation in neural sensory processing by deletion size, age, and sex in Phelan-McDermid syndrome
Source: J Neurodev Disord. 2025 Aug 25;17:51. doi: 10.1186/s11689-025-09642-4 (PMC12376477; doi:10.1186/s11689-025-09642-4)
Supplement: Supplementary file 1 — Supplementary Material 1 [file 11689_2025_9642_MOESM1_ESM.docx]

**Supplementary Materials**

**System and Trial Effects:**

**System**

*Frontal.* EEG system was a significant predictor of P50 and N1 latency by stimulus, in which the change in latency from stimulus 1 to 2 was smaller for EGI than BioSemi. Additionally, BioSemi data generally had higher gamma power than EGI data (Table 8). An age*system interaction suggested that for the youngest individuals, delta, theta, alpha, beta, and gamma were larger for BioSemi vs. EGI systems and vice versa for the oldest individuals (Table 8). These discrepancies may be explained by system differences in mean age, in which more BioSemi data came from younger participants compared to EGI (Table 1). A sex*system interaction suggested that the difference between males and females in delta and theta power was larger for data measured with BioSemi caps than EGI caps. A group*system interaction suggested that the difference between alpha power for PMS vs. TD was greater for data collected from BioSemi caps vs. EGI caps. A system*trials interaction showed that P50 latency was longer and delta power was stronger for BioSemi than EGI as trial counts increased. Importantly, EGI and BioSemi differed in mean trials, which may drive this trial*system effect (Table 1).

In the PMS only analyses, a stimulus by system and age by system interaction showed that BioSemi had shorter P50 latencies than EGI with age as well as for stimulus 2 vs. stimulus 1. BioSemi also had more alpha and gamma than EGI. Older participants had less gamma and more alpha-beta ITC for BioSemi than EGI; females had more alpha-beta ITC and males had less alpha-beta ITC for BioSemi than EGI. Lastly, beta-gamma ITC was lower for BioSemi than EGI for the highest but not lowest trial counts.

*Whole-Head.* Across PMS and TD, there were no main effects of system for ERPs; however, P50 latencies changed more from stimulus 1 to 2 for EGI than for BioSemi. System also had strong effects on all frequency bands except gamma such that BioSemi had more power than EGI. An age*system interaction showed that P50 GFP, alpha, beta, and gamma power were stronger for older individuals with EGI data than with BioSemi data.

In PMS only analyses, P50 latencies were shorter and N1 latencies were longer for BioSemi. BioSemi particularly had longer N1 latencies than EGI for larger deletion sizes, higher trial counts, males, and stimulus 1. BioSemi also had more gamma power within PMS, and older participants generally had less beta power for BioSemi than EGI, which again may be the result of imbalanced samples (e.g., sex).

In general, differences between BioSemi and EGI may be caused by site rather than system effects. For instance, twice as many subjects were recorded using EGI systems than BioSemi. EGI also had twice as many males, females, TD, and PMS as BioSemi, which could account for the statistical differences between the systems, particularly in group*system and sex*system interactions (Table 1). Future research should opt for a single system to minimize the possibility of data discrepancies due to system effects. If multiple systems must be used, data from these systems should be matched across important variables. Additionally, different EEG systems often come with different software and default settings. In this study, EGI sites used E-Prime, while BioSemi used Presentation. As an example of potential software-induced differences, E-Prime may have created onset delays for EGI systems, which could have led to system-based differences in final latency values.

**Trials**

*Frontal.* Across PMS and TD, increases in trial count corresponded to smaller N1 latencies as well as larger decreases in delta, theta, alpha, and beta power for PMS compared to controls. This may suggest that PMS behaviors leading to greater data artifact (and thus fewer trials) are associated with higher delta through beta power.

In PMS only analyses, for every unit decrease in trial count, N1 amplitudes decreased and N2 latencies increased more with deletion size. The opposite was true when trial counts were high. This finding may indicate that trial counts are more consistent with cognitive function for individuals with larger deletion sizes. For time-frequency variables, gamma generally decreased, and alpha-beta and beta-gamma ITC generally increased with trial count for individuals with PMS. Lastly, as trial counts increased, N2 amplitudes and beta-gamma ITC generally took on more positive values with age, which is unusual for N2 but expected for ITC. Since N2 weakens through adulthood, it is possible that the unexpected age by trials interaction is indicative of developmental differences in N2 amplitudes.

*Whole-Head.* Across PMS and TD, P2 GFP decreased with trial count. P50 GFP had a sex by trials interaction such that females had weaker peaks than males as trials increased. A group by trials interaction showed that delta through gamma power decreased more for PMS compared to TD as trials increased. The difference between PMS and TD was larger when there were fewer trials, which is consistent with the idea that individuals with more neurological impairment also have more behavioral impairment that contributes to trial artifact.

For PMS only analyses, a main effect of trials showed smaller P2 and N2 GFP amplitudes as trials increased, which may suggest that P2 and N2 are better characterized by frontal electrodes for individuals with less impairment. Additionally, greater trial counts corresponded to less delta, theta, alpha, and beta power, suggesting higher power for individuals with the fewest trials. An age*trials interaction suggested that as trials increased, theta, alpha, and beta decreased, and beta-gamma ITC increased more so for older participants than young ones.

**Supplemental Table 1.** List of psychoactive drugs reported for individuals with medication data in the PMS cohort. Of the 16 individuals with medication data (10 females, 6 males), 11 were prescribed psychoactive medication. Among these 11 individuals, 5 (5 females, 0 males) were prescribed one psychoactive medication, and 6 (4 females, 2 males) were prescribed multiple psychoactive medications.

| **Medication Class** | **Medication List** | **No. Subjects** | **Sex** |
| --- | --- | --- | --- |
| Antidepressants | Atomoxetine | 1 | F |
|  | Trazodone | 2 | F, F |
|  | Imipramine | 1 | F |
|  | Prozac | 1 | M |
| Benzodiazepines | Diastat | 1 | M |
|  | Bromazepam | 1 | M |
|  | Clonazepam | 1 | F |
| Alpha-2 Agonists (ADHD) | Intuniv | 1 | F |
|  | Clonodine | 1 | F |
| Anticholinergics | Robinul | 1 | F |
|  | Benztropine | 1 | F |
| Anxiolytics | Buspirone | 1 | F |
| Anticonvulsants | Lanictine | 1 | F |
|  | Keppra | 1 | F |
|  | Lamotrigine | 1 | F |
|  | Klononpin | 1 | F |
|  | Lamictal | 2 | M, F |
|  | Depakote | 2 | M, F |
|  | Clonidine | 1 | F |
|  | Onfi | 1 | F |
| Stimulants | Adderall | 2 | F, F |
|  | Focalin | 1 | M |
|  | Methylphenidate | 1 | F |
| Antipsychotics | Seroquel | 2 | M, F |
|  | Risperdone | 2 | M, F |
| Sedatives | Gabapentin | 1 | F |

**Supplemental Table 2.** Non-parametric exact Wilcoxon-Mann-Whitney tests of EEG outcomes between PMS and TD. These analyses are stratified by auditory stimulus (first and second) and performed for frontal and whole-head electrode sets in the full sample (N = 37; 21 PMS, 16 TD) and in the age 8-12 restricted sample (N = 23; 11 PMS, 12 TD). Reported values are Hodges-Lehmann estimates of the median difference between groups. The shading highlights potentially clinically relevant cells in which p < 0.25. ~p < .10, *p < .05, **p < .01, ***p < .001. P-values are exact and two-tailed. L, latency; ITC, intertrial coherence. Negative values are lower for TD and positive values are higher for TD vs. PMS.

|  | **Frontal** | | | | **Whole-Head** | | | |
| --- | --- | --- | --- | --- | --- | --- | --- | --- |
|  | **First Stimulus** | | **Second Stimulus** | | **First Stimulus** | | **Second Stimulus** | |
|  | Full Sample | Age-Restricted | Full Sample | Age-Restricted | Full Sample | Age-Restricted | Full Sample | Age-Restricted |
| **P50 (μV)** | 0.3 (-0.4, 0.9) | 0.1 (-0.9, 1.3) | 0.1 (-0.5, 0.6) | -0.1 (-0.8, 0.8) | *0.4 (0.0, 0.9) | 0.1 (-0.7, 0.7) | 0.1 (-0.1, 0.4) | 0.0 (-0.4, 0.4) |
| **N1 (μV)** | -0.1 (-0.8, 0.4) | 0.1 (-0.5, 0.7) | -0.2 (-0.5, 0.2) | -0.1 (-0.5, 0.5) | ~0.5 (-0.1, 1.0) | 0.0 (-0.7, 0.8) | 0.1 (-0.2, 0.4) | -0.1 (-0.5, 0.4) |
| **P2 (μV)** | 0.5 (-0.1, 1.2) | 0.1 (-0.7, 0.9) | 0.1 (-0.4, 0.6) | -0.2 (-0.9, 0.5) | *0.6 (0.1, 1.0) | 0.1 (-0.5, 0.8) | 0.2 (-0.1, 0.5) | 0.0 (-0.4, 0.5) |
| **N2 (μV)** | -0.4 (-0.9, 0.1) | -0.3 (-1.0, 0.5) | -0.3 (-0.7, 0.1) | -0.1 (-0.8, 0.5) | ~0.4 (-0.0, 0.8) | 0.2 (-0.2, 0.7) | 0.2 (-0.0, 0.5) | -0.0 (-0.4, 0.4) |
| **P50L (ms)** | -8 (-22, 4) | -6 (-26, 6) | -2 (-18, 16) | -6 (-26, 16) | -10 (-22, 4) | ~-18 (-32, 4) | -5 (-26, 10) | 2 (-20, 32) |
| **N1L (ms)** | *-30 (-70, 0) | -23 (-78, 4) | 6 (-18, 36) | 6 (-20, 44) | 21 (-4, 50) | ~36 (0, 68) | 4 (-18, 32) | -0 (-32, 28) |
| **P2L (ms)** | -5 (-36, 20) | 12 (-40, 40) | 24 (0, 46) | ~30 (0, 52) | 18 (-4, 42) | **42 (14, 72) | 2 (-24, 44) | 3 (-38, 58) |
| **N2L (ms)** | -23 (-62, 24) | -2 (-56, 42) | 24 (-10, 54) | 15 (-28, 58) | -4 (-36, 22) | -12 (-58, 24) | -14 (-50, 18) | -10 (-50, 30) |
| **Delta (μV^2^)** | 1.7 (-0.1, 4.3) | 0.4 (-1.3, 3.9) | 1.7 (-0.4, 4.0) | 0.7 (-1.2, 4.4) | 1.3 (-0.6, 3.2) | 0.9 (-1.2, 2.8) | 0.8 (-1.2, 2.8) | 0.5 (-1.4, 2.4) |
| **Theta (μV^2^)** | 1.1 (-1.4, 4.0) | -0.0 (-2.1, 3.3) | 1.3 (-1.1, 4.0) | -0.1 (-1.9, 4.1) | ~1.5 (-0.2, 3.5) | 1.0 (-0.8, 2.9) | 1.1 (-0.7, 3.0) | 0.6 (-0.8, 2.6) |
| **Alpha (μV^2^)** | 1.1 (-1.1, 3.3) | 0.0 (-2.4, 3.5) | 1.0 (-1.2, 3.5) | 0.3 (-2.4, 3.7) | ~1.5 (-0.4, 3.0) | 0.9 (-0.9, 2.5) | 1.3 (-0.5, 2.9) | 0.4 (-1.0, 2.5) |
| **Beta (μV^2^)** | 0.1 (-2.0, 2.1) | -1.3 (-3.3, 3.6) | -0.0 (-2.2, 2.1) | -1.3 (-3.4, 3.0) | 0.5 (-1.0, 1.8) | -0.0 (-1.6, 1.6) | 0.4 (-1.3, 1.7) | -0.5 (-2.0, 1.2) |
| **Gamma (μV^2^)** | 0.2 (-2.3, 3.1) | 0.4 (-2.8, 4.2) | 0.5 (-2.3, 3.1) | 0.5 (-2.7, 4.2) | -1.0 (-2.5, 0.8) | -0.6 (-2.7, 1.9) | -0.9 (-2.2, 0.9) | -0.5 (-2.7, 1.7) |
| **Beta-Gamma (ITC)** | *0.0 (0.0, 0.1) | *0.1 (0.0, 0.1) | 0.0 (-0.0, 0.1) | ~0.0 (-0.0, 0.1) | ~0.0 (-0.0, 0.0) | 0.0 (-0.0, 0.0) | -0.0 (-0.0, 0.0) | -0.0 (-0.0, 0.0) |
| **Alpha-Beta (ITC)** | ~0.1 (-0.0, 0.1) | *0.1 (0.0, 0.2) | -0.0 (-0.0, 0.0) | -0.0 (-0.0, 0.1) | ~0.0 (-0.0, 0.0) | 0.0 (-0.0, 0.0) | 0.0 (-0.0, 0.0) | 0.0 (-0.0, 0.0) |
| **Delta-Theta (ITC)** | 0.0 (-0.0, 0.1) | 0.1 (-0.0, 0.2) | 0.0 (-0.1, 0.1) | 0.0 (-0.1, 0.1) | 0.0 (-0.0, 0.0) | 0.0 (-0.0, 0.1) | 0.0 (-0.0, 0.0) | *0.0 (0.0, 0.1) |


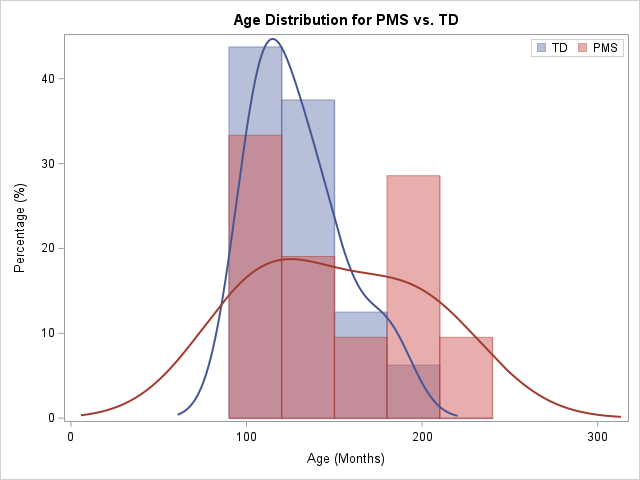


**Supplemental Figure 1.** The distribution of age by group (PMS vs. TD).


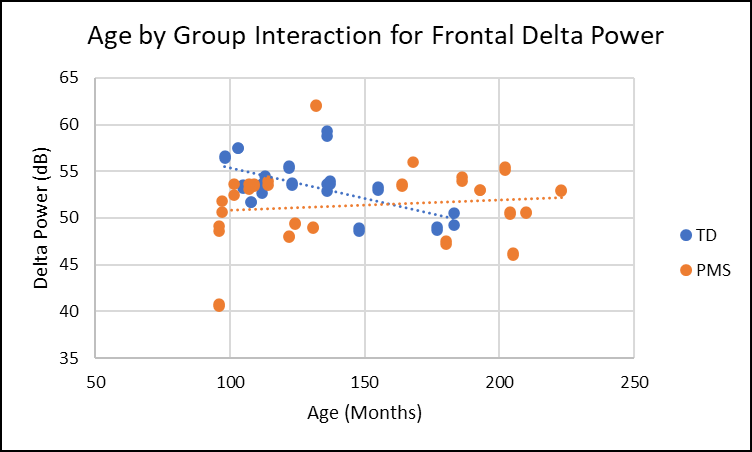


**Supplemental Figure 2.** Example age by group interaction for frontal delta power (Table 5). The data plotted below were not adjusted for other model variables.


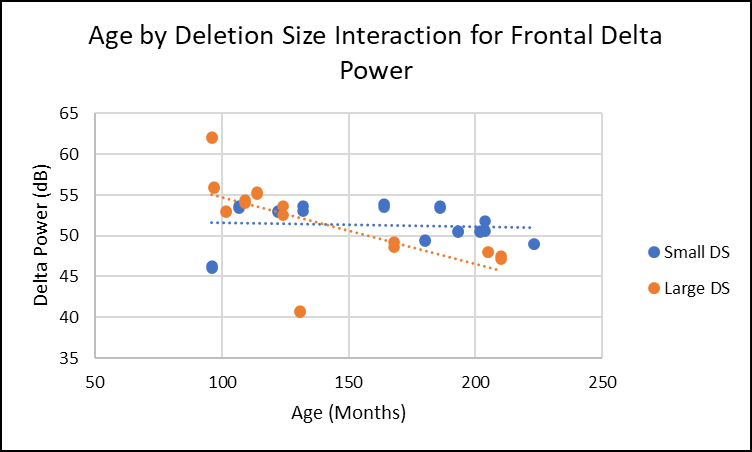


**Supplemental Figure 3.** Example age by deletion size interaction for frontal delta power (Table 8). The data plotted below were not adjusted for other model variables. A median split was applied to deletion size to assist with visualization of this interaction.


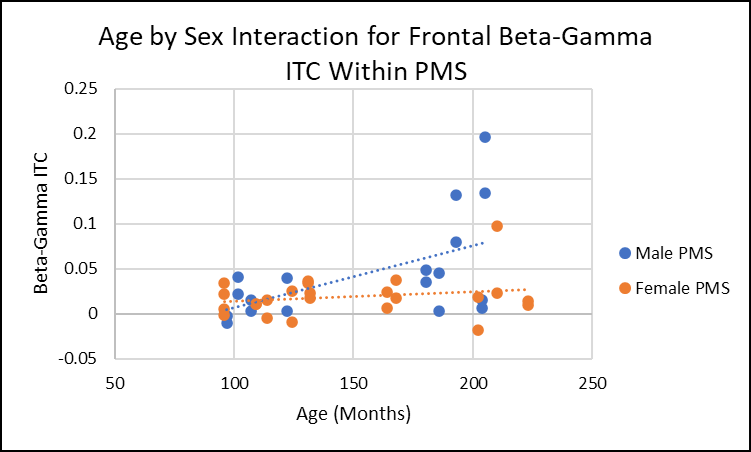


**Supplemental Figure 4.** Example age by sex interaction for beta-gamma ITC among individuals with PMS (Table 8). The data plotted below were not adjusted for other model variables.


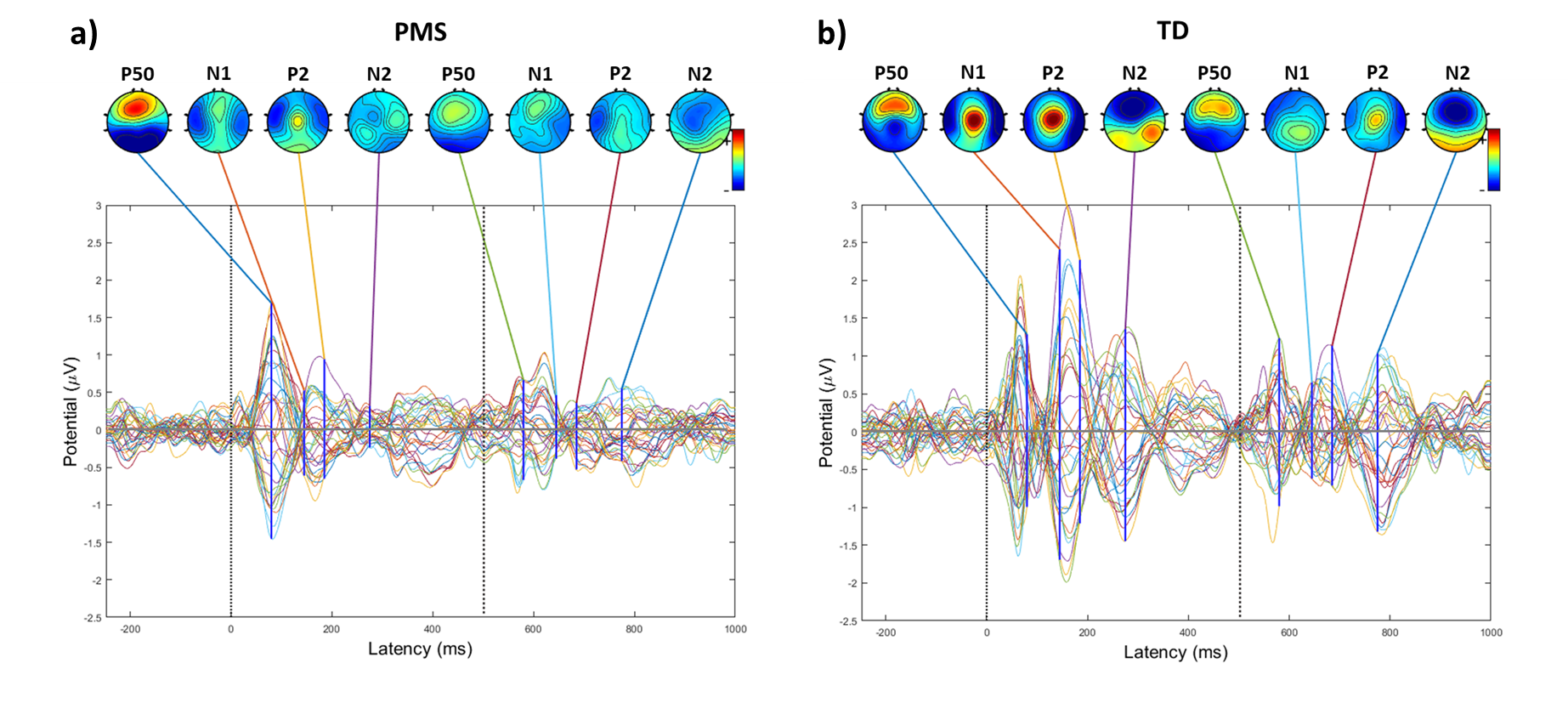
**Supplemental Figure 5.** Topoplots of grand average ERP activity among a) typically developing individuals and 2) those with PMS. Each topoplot represents whole-head brain activity at the midpoint of our time windows for the P50, N1, P2, and N2.
